# Weak Adoption and Performance of Hepatitis B Birth-Dose Vaccination Programs in Africa: Time to Consider Systems Complexity?—A Scoping Review

**DOI:** 10.3390/tropicalmed8100474

**Published:** 2023-10-16

**Authors:** Tasneem Solomon-Rakiep, Jill Olivier, Edina Amponsah-Dacosta

**Affiliations:** 1 Health Policy and Systems Division, School of Public Health, Faculty of Health Sciences, University of Cape Town, Observatory, Cape Town 7925, South Africa; jill.olivier@uct.ac.za; 2 Vaccines for Africa Initiative, School of Public Health, Faculty of Health Sciences, University of Cape Town, Observatory, Cape Town 7925, South Africa

**Keywords:** Africa, birth-dose, hepatitis B, health systems, maternal and child health, vaccine

## Abstract

The persistent burden of chronic hepatitis B among ≤5-year-old children in Africa suggests missed opportunities for controlling mother-to-child transmission (MTCT) of the hepatitis B virus (HBV). This scoping review maps the evidence base on the risk of HBV MTCT, the status of HBV MTCT mitigation strategies including hepatitis B birth-dose vaccination, and the role of systems complexity on the suboptimal adoption and performance of hepatitis B birth-dose vaccination programs in Africa. Overall, 88 peer-reviewed and grey literature sources published between 2000–2022 were included in this review. The growing evidence base consistently argues for a heightened risk of HBV MTCT amidst the HIV co-epidemic in the region. Without universal HBV screening programs integrated within broader antenatal care services, current selective hepatitis B birth-dose vaccination is unlikely to effectively interrupt HBV MTCT. We underscore critical health systems-related barriers to universal adoption and optimal performance of hepatitis B birth-dose vaccination programs in the region. To better conceptualize the role of complexity and system-wide effects on the observed performance of the program, we propose an adapted systems-based logic model. Ultimately, exploring contextualized complex systems approaches to scaling-up universal hepatitis B birth-dose vaccination programs should form an integral part of the regional research agenda.

## 1. Introduction

Chronic hepatitis B caused by persistent infection with the hepatitis B virus (HBV) is a major public health threat in endemic regions like the World Health Organization (WHO) Africa region [1]. Chronic infection with HBV poses a 15–25% lifetime risk of acquiring liver cirrhosis or hepatocellular carcinoma [1,2]. Thus, in the absence of interventions, ~90% of babies born to mothers testing positive for the hepatitis B surface (HBsAg) or e (HBeAg) antigens will develop chronic infection, raising significant global public health concern [1]. Debates on the leading route of transmission driving the epidemic in Africa have previously favored horizontal transmission in early childhood [3,4]. However, a growing body of evidence now suggests an epidemiological shift towards HBV mother-to-child-transmission (MTCT) or vertical transmission [2,5,6,7,8,9,10,11,12,13,14,15]. This cannot be addressed outside of the disproportionate HIV epidemic faced by the WHO Africa region, home to 66% (25 million) of the global total of people living with HIV [16,17,18]. Persons living with HIV have a 40% higher risk of acquiring HBV infection, and HBV–HIV co-infections are associated with a higher likelihood of HBV MTCT compared to HBV mono-infection [19,20].

While the WHO Western Pacific region has more chronic HBV carriers (115 million or 5.9% [95% Uncertainty Interval (UI) 4.9–7.3] vs. 82 million or 7.5% [95% UI 5.7–10.5] of the population in the WHO Africa region), the highest proportion of children younger than 5 years of age living with HBV are in Africa, estimated at 2.5%, which surpasses the global prevalence of 0.9% [95% UI 0.7–1.6] [16,17]. This suggests that the WHO Africa region is on course to having one of the largest populations of chronic HBV carriers compared to any other region without urgent and intensive public health intervention. Fortunately, chronic hepatitis B is entirely vaccine preventable [1]. Universal hepatitis B infant vaccination has been adopted in all 47 WHO Africa member states since the early 1990s, maintaining relatively good coverage rates (72% in 2022) [21]. Despite this, the continued burden of chronic hepatitis B among children younger than 5 years of age suggests missed opportunities for controlling HBV MTCT in the WHO Africa region.

Universal hepatitis B birth-dose vaccination is recommended for the prevention of HBV MTCT and has been endorsed by the WHO for all member states since 2009 [1,22,23,24]. It forms an integral part of the World Health Assembly’s *Global Health Sector Strategy (GHSS) on Viral Hepatitis 2016–2021*, which aims to eliminate viral hepatitis as a global public health threat by 2030 [25]. Globally, 111 of 194 WHO member states have adopted universal hepatitis B birth-dose vaccination programs [26,27] with coverage rates reaching 45% in 2022 [21]. Despite the commitment of the WHO Africa regional office to the GHSS goal [28], the adoption of universal hepatitis B birth-dose vaccination programs has been slow with only 15 of 47 member states adopting it to date [29,30]. Furthermore, dismal coverage of the hepatitis B birth-dose vaccine in the WHO Africa region (18% in 2022) suggests significant programmatic challenges [21].

Identifying barriers to the adoption and optimal performance of hepatitis B birth-dose vaccination programs alone, though helpful, is unlikely to inform the development of sustainable solutions. To achieve whole-of-systems gains, it has been suggested that critical attention should be given to the broader health systems delivering health interventions [31,32]. Because health systems can adapt to changes in the local environment and are composed of other complex systems (i.e., people at the center of the health system), they are recognized as complex systems [33,34]. Acknowledging the influence of health systems complexity may assist in strengthening the capacity to support interventions like hepatitis B birth-dose vaccination. Interventions themselves can also introduce some degree of complexity when characterized by multiple interacting components, limited degree of flexibility of implementation, non-linear causal pathways, and feedback loops [35,36,37,38]. Some argue, therefore, that key consideration should be given to the complex interaction between interventions and the health systems they are embedded in [33,39]. We posit that research enquiries, including evidence syntheses, adopting complex systems perspectives could uncover richer explanations for the suboptimal performance of hepatitis B birth-dose vaccination programs in the WHO Africa region [36,38].

## 2. Materials and Methods

### 2.1. Objectives of the Review

The overarching purpose of this scoping review was to better understand the breadth and depth of evidence on the status of hepatitis B birth-dose vaccination programs in the WHO Africa region and explore the potential for further research enquiry into the role of systems complexity. For this purpose, we address the following objectives:

- To describe current knowledge on the risk of HBV MTCT in the WHO Africa region;
- To describe the status of HBV MTCT mitigation strategies including hepatitis B birth-dose vaccination programs;
- To explore health systems’ capacity to support hepatitis B birth-dose vaccination programs in the WHO Africa region.

### 2.2. Methods

This scoping review was conducted in alignment with the Joanna Briggs Institute guidelines on scoping reviews together with the PRISMA extension for Scoping Reviews (PRISMA-ScR) checklist (see Table A1 in the Appendix A) [40,41].

Several electronic databases and platforms were consulted for peer-reviewed literature, namely, PubMed, Scopus, Web of Science, and EBSCOhost (Academic Search Premier, Africa-Wide Information, CINAHL, Health Source: Nursing/Academic Edition, and APA PsycInfo). Additionally, grey literature was sourced from organizational websites like WHO (http://www.who.int/ (accessed on 15 August 2022)), United Nations International Children’s Fund (UNICEF, https://www.unicef.org/ (accessed on 15 August 2022)) and Gavi, the Vaccine Alliance (http://www.gavi.org/ (accessed on 15 August 2022)). Supplementary searches for peer-reviewed and grey literature were sourced by reviewing bibliographies and performing additional internet searches on Google Scholar. Developed with guidance from an information specialist, the search strategy included synonyms of keywords and terms like hepatitis B, hepatitis B vaccines, birth dose, and birth dose vaccine (Table S1 in the Supplementary Materials).

Only relevant literature published in English was considered. To map the context, progress, and challenges of adopting hepatitis B birth-dose vaccination programs over time, relevant literature published within the last 22 years (2000–2022) was sourced. This spans the period before and after the WHO recommendation on universal hepatitis B birth-dose vaccination in 2009. All search yields were imported to Mendeley Reference Manager^®^ [42] and, after removal of duplicate records, exported to Rayyan^®^ [43]. Here, titles, abstracts, and full texts were screened against the inclusion and exclusion criteria, and eligible articles selected for this review. Data charting involved extraction of information like author name, year of publication, title, study setting, study design, and main outcomes from full-text articles, which were then reported as narrative summaries.

## 3. Results

**Figure 1:**
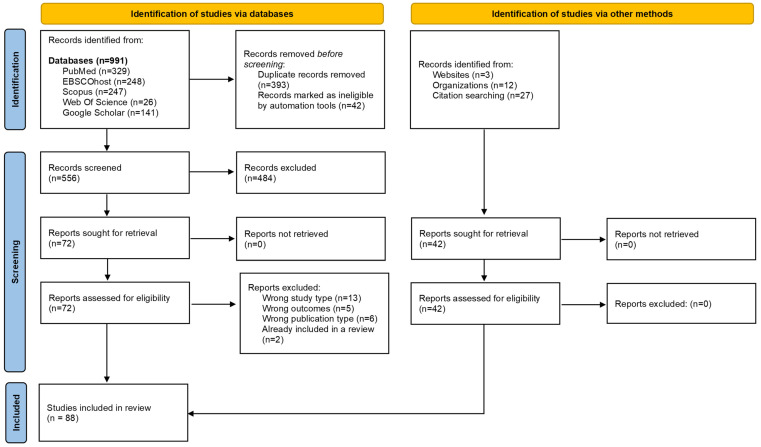
PRISMA flow diagram of literature search, screening, and selection process [51].

A total of 991 records were retrieved from all the databases searched. After deduplication and screening of abstracts, titles, and full-text, 72 unique records were identified. Of the 72 records, 46 reported on relevant research conducted in the WHO Africa region. Additional published literature sources were identified through supplementary searches and judged to be eligible for inclusion. Overall, 88 literature sources were included in this scoping review (Figure 1). These varied across study types and included quantitative and qualitative empirical studies, scoping and systematic reviews, commentaries and expert opinions, international guidelines, organizational webpages, reports, and theses (Table S2). The findings of this scoping review are organized and reported under three key themes, namely, (i) current knowledge on the risk of HBV MTCT, (ii) status of HBV MTCT mitigation strategies, and (iii) health systems’ capacity to support hepatitis B birth-dose vaccination in the WHO Africa region.

### 3.1. Current Knowledge on the Risk of HBV MTCT in the WHO Africa Region

Areas of high endemicity are defined as having an HBsAg prevalence of ≥8%, moderate endemicity between 2–7% and low endemicity of <2% in the general population [2,6]. In Africa, an estimated HBsAg prevalence of 7.5% is recorded, and Africa is considered home to approximately 28% of the world’s 296 million chronic carriers [16,17]. Prior to regional adoption of routine hepatitis B vaccination, >95% of all infections occurring in infancy were associated with horizontal transmission [3]. In recent years, a growing risk of vertical transmission (>5%) has been observed and is cited to be influenced by the HIV epidemic, as subsequently discussed in Section 3.1.2 [3,19]. Despite this, strategies employed to control the burden of hepatitis B depend almost solely on the 1992 WHO recommendation of universal hepatitis B infant vaccination commencing at 4 or 6 weeks of life [5]. In the absence of strategies targeting the interruption of vertical transmission, the cycle of chronic infection continues to fuel the morbidity and mortality [2,15]. The *immaturity* of the neonatal immune system increases the risk of viral replication and is suggested as the reason for delayed clearance of HBeAg [44], whereas acute infections in immunocompetent adults are likely to be cleared [15]. In a meta-analysis of 15 articles investigating HBV infection among women in sub-Saharan Africa, a total of 14,239 women were screened for HBsAg and a further 951 for HBeAg [45]. Among these studies, HBeAg positivity was shown to increase the risk of vertical transmission to 38.3% compared to 4.8% in HBeAg negative women [45]. Therefore, assessing the increased risk of HBV MTCT, the influence of HIV co-infection, and the strategies available for effective prevention of HBV MTCT in Africa is essential.

#### 3.1.1. Growing Evidence on the Risk of HBV MTCT

It is established that HBV MTCT depends on the presence of increased maternal infectiousness, correlating with HBeAg positivity or high HBV DNA viral loads [1]. Historically, women of child-bearing age in Africa are considered to have a relatively low prevalence of HBeAg (0.5–3.5%) compared to that in South and South-East Asia (78%) [5,6,46]. Growing evidence on the risk of HBV MTCT in the African region now suggests moderate to high endemicity of HBV infection among pregnant women and women of child-bearing age [2,5,6,47]. A recent review by Breakwell et al. identified 75 studies across 18 countries in Africa that report the HBeAg prevalence rates among HBsAg-positive pregnant women, ranging from 3.3% in Zimbabwe to 28.5% in Nigeria [2]. Studies assessing perinatal transmission in mother–child pairs by testing the HBsAg and HBeAg prevalence in mothers and their offspring have also found high rates of paired positivity [2,5,6], and more so in those mothers testing positive for HBeAg or with high HBV DNA levels [2,5,6]. It is worth noting that without appropriate intervention, vertical transmission is still possible among 2–10% of HBeAg negative pregnant women within the region [5].

In a 2016 situational report compiled by the WHO Africa regional office, the scarcity of evidence on chronic HBV infection and the risk of perinatal transmission in Africa were identified by 6 countries as one of the barriers to hepatitis B birth-dose introduction [48]. Since then, the knowledge base has developed and consistently points to a growing risk of vertical transmission within the region [7,8,9,10,11,12,13,14,15,49], as presented in Table 1. All studies report moderate to high HBV prevalence among pregnant or postpartum women. This is demonstrated by the high HBsAg prevalence among pregnant women in countries like Ethiopia (6.9%), Cameroon (7.7%), The Gambia (9.2%), South Sudan (11%), and Uganda (11.8%) [7,8,9,10,11]. In comparison, moderate HBsAg prevalence rates have been reported among pregnant women in countries like the Republic of Congo (2.7%), Tanzania (3.9%), South Africa (4.5%), and Burkina Faso (4.8%) [12,13,14,15]. Evidently, substantial variations exist in the burden of disease across countries, with the highest HBsAg prevalence rates noted in the Central and West African regions [5,50].

#### 3.1.2. HIV–HBV Co-Infection and the Increased Risk of HBV MTCT

Accompanying the high burden of HBV infections in the region is the simultaneous burden of HIV infection [20]. It is estimated that 18 million HIV positive women reside in Africa, of whom the majority are of child-bearing age [1]. Given that those who are co-infected with HIV have higher rates of chronicity and occult HBV infection [52], a greater prevalence of HBeAg positivity and higher HBV DNA viral loads [4,19], and are subject to frequent reactivation of latent HBV infections [19], the risk of HBV MTCT in HIV co-infected pregnant women is increased compared to that in their HBV mono-infected counterparts [19]. A global review of HIV–HBV co-infections cited a 4.6% midpoint prevalence of HBsAg from 23 studies involving pregnant women [52]. Several studies in Africa have demonstrated the significant risk of vertical transmission of HBV in both HIV-seropositive and -negative populations [19,23,53]. These studies report HBsAg positivity rates ranging from 2.1–3.4% among HIV-seropositive and 0.4–3.8% in HIV-negative pregnant women [19,23,53]. Further to this, these studies report comparable rates of HBeAg positivity among pregnant women living with (18.9% [19], 30% [23]) and without (17.1% [19], 37.6% [23]) HIV. It is estimated that around 10% of HBV-infected pregnant women are HBeAg positive regardless of HIV serostatus, although significant disparities exist in the risk of HBV MTCT within the region [47]. This has been demonstrated by the high HBeAg positivity rates among HBsAg-positive pregnant women across the region like the Democratic Republic of Congo (10%), Burkina Faso (11.5%), Uganda (14.9%), South Africa (16.6%), and Cameroon (26.1%) [7,10,13,14,15]. In two South African studies comparing HBsAg-positive pregnant women stratified by HIV status, no significant difference in HBeAg positivity between either group was found [19,23]. The widespread availability of HBV-active antiviral therapy for those living with HIV offers a plausible explanation for the similarities in prevalence of HBeAg positivity between HIV-positive and -negative pregnant women [1,20]. Studies in Uganda, Senegal, Zambia, and Cameroon have demonstrated the protective effect of HBV-active antiviral therapy in preventing HBV MTCT [7,49,53,54]. These available antiviral therapies like lamivudine, telbivudine, tenofovir, and entecavir have largely been proven effective and safe in reducing HBV viral loads in pregnant women [1]. Unfortunately, access and procurement of these medications have been limited to HIV-positive populations leaving HBV mono-infected pregnant women at greater risk for vertical transmission [1,4,20].

**Table 1:** Growing evidence on the seroprevalence and risk of HBV MTCT in the WHO Africa region.

Study No.	Author, Year	Setting	Study Design	Study Period	Population and Population Size	Summary of Key Findings
1	Rashid et al., 2014 [12]	Tanzania	Cross-sectional	August–September 2010	310 Pregnant women	Of 310 pregnant women, 3.9% tested HBsAg positive Of these, none tested positive for HBeAg 9.7% (30/310) tested positive for HIV with 25% of these women co-infected with HBV All HBsAg-positive women tested negative for markers of acute infection suggesting chronic carriage
2	Bayo et al., 2014 [10]	Uganda	Cross-sectional	September 2012–January 2013	397 Pregnant women	11.8% (47/397) tested positive for HBsAg Of these, 14.9% (7/47) tested HBeAg positive An HIV positivity rate of 9.3% was found among pregnant women but no significant association between HIV and HBV seropositivity Those ≤20 years old were 2.5 times more likely to test positive for HBsAg
3	Howell et al., 2014 [5]	Sub-Saharan Africa	Literature Review	Publications between 1995–2013	60,177 Pregnant women and women of childbearing age	Regional HBsAg prevalence ranges from 2.4–25% Regional HBeAg prevalence ranges from 1–35% HBsAg prevalence within this population is highest in West Africa
					528 Mother–child pairs	Burkina Faso: 37% of 35 unvaccinated infants born to HBsAg-positive mothers tested HBsAg positive. Among these, 53.3% were from HBeAg-positive mothers vs. 27% from HBeAg-negative mothers Ghana: 8% of unvaccinated infants born to 219 HBsAg-positive mothers tested HBsAg positive. 3% (6/219) of the HBsAg-positive mothers transmitted HBV despite being HBeAg negative Tanzania: Among 62 unvaccinated infants, 8% born to HBsAg-positive/HBeAg-positive mothers tested HBsAg positive compared to 2% of infants born to HBsAg-positive/HBeAg-negative mothers at 8 months of age Nigeria: 72% of 50 unvaccinated infants born to HBsAg-positive mothers had detectable HBV DNA in infant cord blood but only 24% of these tested HBsAg positive Cote d’Ivoire: In a cohort of 60, 32.5% of hepatitis B birth-dose vaccinated infants born to HBsAg-positive mothers tested HBsAg positive on cord blood. 66.7% of these infants were born to HBeAg-positive mothers and 26.9% were born to HBeAg-negative mothers. No follow-up for vaccine effectiveness performed Malawi: among 102 vaccinated infants born to HIV–HBV co-infected mothers, 9.8% of infants tested HBsAg positive
4	Sadoh et al., 2014 [6]	Nigeria	Literature Review	Period not specified. References range from 1988–2013	Pregnant women and women of childbearing age	Prevalence of HBsAg ranges from 8.3–12.8% Prevalence of HBeAg ranges from 7.9–62.5%
					Mother–child pairs (Size of population not consistently reported)	Two studies expressed a MTCT rate of 42.86% and 53.3%, respectively In a third study, an HBsAg positivity rate of 13.2% correlated with a 6.9% HBsAg positivity rate among children. Further, 85% of seropositive children were born to HBsAg-positive mothers while 77.7% HBeAg-positive mothers had seropositive children In a fourth study, 29.4% of infants who presented for their hepatitis B birth-dose vaccination were HBsAg positive Most infants did not receive their vaccine within 24 h post-birth
5	Umare et al., 2016 [9]	Ethiopia	Cross-sectional	March–May 2015	338 Pregnant women	Overall, HBsAg positivity rate was 6.9% HBsAg prevalence was 8% in age group 20–24 years old The highest HBsAg prevalence rate (37.5%) was recorded among pregnant women who were health care workers
6	Breakwell et al., 2017 [2]	WHO AFRO	Literature review	January 1995–October 2016	Median range 269–2244 Pregnant women across 75 studies	Median prevalence of HBsAg ranging from 1.9% in Madagascar to 16.2% in Niger Median prevalence of HBeAg ranging from 3.3% in Zimbabwe to 28.5% in Nigeria
				WHO/UNICEF Monitoring data updated to year 2016	143 Mother–child pairs	Cote d’Ivoire: 38% of 24 infants born to HBsAg-positive/HBeAg-positive mothers tested HBsAg positive at 6 weeks of life compared to 0% of infants born to HBsAg-positive/HBeAg-negative mothers Ghana: 5.2% of 97 infants born to HBsAg-positive mothers tested HBV DNA–positive at 2 weeks of life Burkina Faso: 32% of 22 infants born to HBsAg-positive/HBeAg-negative mothers tested HBsAg positive within 24 h of birth, compared to 29% of infants born to HBsAg-positive/HBeAg-positive mothers
7	Chotun et al., 2017 [15]	South Africa	Prospective cohort	June–November 2014	134 Pregnant women	4.5% (6/134) tested positive for HBsAg Of those HBsAg positive, 16.6% (1/6) tested HBeAg positive 33.3% (2/6) of the HBsAg-positive women had elevated HBV DNA levels and were initiated on antivirals All women tested negative for markers of acute infection, suggesting chronic infection
					4 Infants	100% of all infants tested (*n* = 4) had undetectable HBV DNA at mean age of 97 days of life All infants had undetectable HBV DNA when followed up at mean age of 328 days of life/7 months
8	Kirbak et al., 2017 [11]	South Sudan	Cross-sectional	December 2012–March 2013	280 Pregnant women	11% (31/280) tested positive for HBsAg 28% of pregnant women had natural immunity, 2% immunity due to vaccination, 36% were indeterminate, and 23% susceptible to infection
9	Seremba et al., 2017 [49]	Uganda	Cross-sectional	July 2012–June 2014	612 Mothers	8.7% (*n* = 53) tested positive for HBsAg Of these women, 18.9% (*n* = 10) also tested positive for HBeAg 11.5% (9/80) of HIV-infected individuals were co-infected with HBV 55.6% of HIV/HBV co-infected pregnant women were on HBV-active antivirals In total, 81.3% (65/80) of HIV-positive mothers were on HBV-active antivirals
					606 Infants	No HBsAg-positive results at 6 weeks of life, on presentation for the first vaccinations HBV MTCT was deemed to not make a substantial contribution to the burden of disease in Uganda
10	Sone et al., 2017 [7]	Cameroon	Prospective cross-sectional	10-month period, year not specified.	298 Pregnant women	7.7% (23/298) of pregnant women were carriers of HBV markers of infection 26.1% (6/23) tested HBsAg positive/HBeAg positive 1% (3/298) HIV/HBV co-infected and on antiviral therapy
				Ethical clearance given in 2014	20 Infants	40% (8/20) of infants born to HBsAg-positive mothers tested positive for HBsAg Of these, 75% (6/8) were HBsAg positive/HBeAg positive 14.3% (2/14) born to HBsAg-positive/HBeAg-negative mothers tested positive for HBsAg 100% of infants born to HIV/HBV co-infected mothers on antivirals tested HBsAg negative 50% (10/20) tested negative for HBsAg with the presence of HBe-antibodies
11	Bittaye et al., 2019 [8]	The Gambia	Cross-sectional	May–July 2015	424 Pregnant women	Prevalence of HBsAg was 9.2% (39/424) Among pregnant women who were likely to be vaccinated, HBsAg prevalence was 2.3% vs. 13.7% among those likely unvaccinated
12	Guingané et al., 2020 [13]	Burkina Faso	Prospective cohort	October 2014–February 2016	1580 Pregnant women	4.8% (75/1580) tested HBsAg positive 11.5% (7/61) of HBsAg positive tested HBeAg positive 7.5% (4/53) of HBsAg-positive samples had elevated HBV DNA levels and initiated antiviral treatment
					40 Infants	Infants received hepatitis B birth-dose vaccination and subsequent hepatitis B infant vaccinations according to recommended schedule Of the infants available for follow up (*n* = 40), 100% tested negative for HBsAg at 7 months of age
13	Thompson et al., 2021 [14]	Democratic Republic of Congo	Cohort	September 2018–February 2019	4016 Pregnant women	2.7% (*n* = 109) tested positive for HBsAg 11% of available samples (*n* = 90) were identified as high risk for MTCT. Of these, 90% were HBeAg positive and 50% had elevated HBV DNA levels 90% of these high-risk pregnant women received antiviral therapy Only 1% (*n* = 1) were co-infected with HIV
					88 Infants	68% (60/88) of infants received hepatitis birth-dose vaccination Of the available sample (*n* = 53) who presented at 24 weeks for follow up testing, 100% tested negative for HBsAg

### 3.2. Status of HBV MTCT Mitigation Strategies in the WHO Africa Region

**Figure 2:**
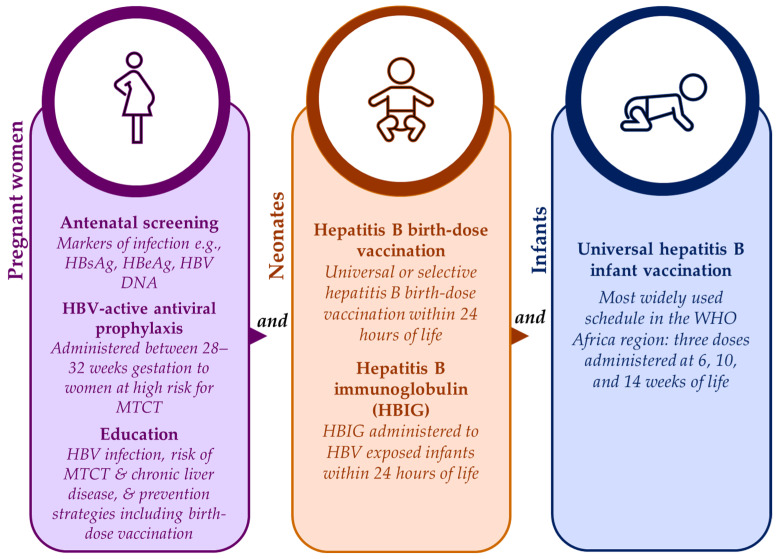
Summary of strategies for the prevention of HBV MTCT [2,4,5,22].

Safe and effective strategies are available for prevention of HBV MTCT to neonates and infants from as early as the in-utero stage, as shown in Figure 2. Antenatal screening has the advantage of identifying those at risk for HBV MTCT who can then be timely linked to appropriate care such as HBV-active antiviral prophylaxis [47]. During antenatal visits, pregnant women should also be provided with information on HBV infection, the lifetime risk for chronic liver disease associated with HBV MTCT, and the prevention strategies available to them and their babies including hepatitis B birth-dose vaccination [23,47]. Unfortunately, for some African countries, antenatal screening for HBV infection can be expensive and impractical if laboratory facilities are not situated close to antenatal clinics [23,47]. Despite this, the feasibility of antenatal screening for HBV infection has been proven in South Africa [15], and cost-effectiveness has been demonstrated in Namibia as part of the national HBV MTCT prevention package [3]. Maximum gains can be achieved if HBV antenatal screening is integrated with existing HIV and syphilis point-of-care testing infrastructure [15,55].

Antiviral prophylaxis offered in the third trimester to at-risk pregnant women has been proven to suppress maternal viral loads and decrease the likelihood of HBV MTCT [4,15,20]. The HBV-active antiviral prophylaxis is safe, effective, and readily available in Africa [20,47]. Despite this, and as alluded to previously, access for HBV mono-infected pregnant women in the region appears limited given that there are no subsidized HBV-active antiviral programs [20,47]. To the contrary, HBV–HIV co-infected pregnant women have access to lifesaving prophylaxis through established HIV treatment programs [20,47]. The fixed combination dosage of HBV-active antivirals (tenofovir, lamivudine/emtricitabine and efavirenz) prescribed for the treatment of HIV is subsidized, leaving resource-constrained settings having to pay higher prices for treatment options (such as tenofovir) for HBV mono-infection [20]. This calls for further interventions at the policy level to improve access to low-cost antiviral prophylaxis for HBV mono-infected pregnant women as part of HBV MTCT prevention strategies [47].

For neonates born to women living with chronic HBV infection, hepatitis B immunoglobulin (HBIG) offers further benefit in preventing HBV MTCT by providing passive immunization to HBV-exposed neonates, particularly when administered within 24 h of birth [3]. According to the international guidelines from key special interest groups like the American and European Associations for the Study of Liver Disease, HBV-exposed infants should receive both HBIG and a birth-dose of the hepatitis B vaccine [56,57]. In high-income countries, the standard of care includes both interventions [20,55]. In low-resource settings, the high cost and the logistics of cold-chain storage and administration of HBIG limit its acceptability as an HBV MTCT prevention strategy [3,4,15,20,47,55].

**Figure 3:**
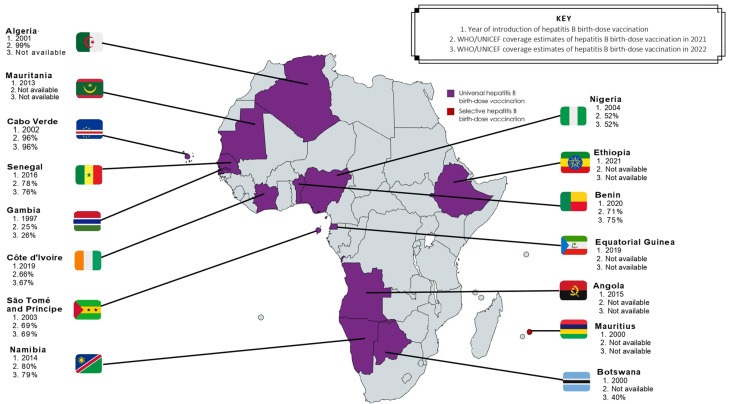
Progress in adoption and coverage of hepatitis B birth-dose vaccination in WHO Africa member states [21,30,62].

Although all strategies have proven effective [23,58], universal hepatitis B birth-dose vaccination has demonstrated suitability for the African context [23] due to both vaccine effectiveness [2,46] and high cost-effectiveness in the region [1,22]. Unlike HBIG, the hepatitis B birth-dose vaccine is stable outside of the cold chain [59], and therefore improves accessibility in low- and middle-income countries (LMICs) [60]. It also presents a feasible opportunity for integration into existing infrastructure like the Expanded Program on Immunization and prevention of HIV MTCT packages [14]. Hence, universal hepatitis B birth-dose vaccination offers an equitable solution to accelerating regional progress towards achieving global elimination of hepatitis B by 2030. Between 75–95% of vertical transmissions can be prevented by vaccinations alone if the birth-dose is followed by completion of at least 3 doses of the hepatitis B vaccine in infancy [22]. The global coverage of hepatitis B birth-dose vaccination has steadily improved from 5% in 2000 to 45% in 2022 [21], although this is well below the GHSS 2030 target of 90% [25]. Among all 6 WHO regions, the coverage rate of the hepatitis B birth-dose vaccine in Africa is significantly dismal at 18%, compared to 80% in the Western Pacific, 65% in the Americas, 58% in South-East Asian, 42% in European, and 32% in the Eastern Mediterranean regions [21]. This suboptimal coverage is independent of the number of African countries who have adopted universal hepatitis B birth-dose vaccination programs [29,30,61]. As shown in Figure 3, only 15 member states currently offer this intervention; Algeria, Angola, Botswana, Cabo Verde, The Gambia, Mauritania, Namibia, Nigeria, São Tomé and Príncipe, Senegal, Cote d’Ivoire, Benin, Equatorial Guinea, and Ethiopia all have universal hepatitis B birth-dose policies in place, whereas Mauritius currently provides selective hepatitis B birth-dose vaccination to HBV-exposed neonates [2,29,30]. It is important to note that while other African countries have not adopted a national universal hepatitis B birth-dose vaccination policy [29], they may provide targeted hepatitis B birth-dose vaccination to infants born to HBeAg-positive mothers in certain sub-regions or health facilities, as per national guidelines for prevention of MTCT or as part of project-based programs like research studies conducted in order to motivate national implementation [14,15]. In this regard, there may be an underestimation of the true coverage of the hepatitis B birth-dose vaccine in Africa compared to what is officially reported to the WHO [21,62]. Without universal and optimal screening programs to identify all at-risk pregnant women, these targeted approaches may encounter significant challenges. Nevertheless, the low implementation of universal hepatitis B birth-dose vaccination within Africa is concerning given the growing risk of HBV MTCT in this region.

Despite the lack of region-wide adoption of hepatitis B birth-dose vaccination, universal hepatitis B infant vaccination programs have been successful at reducing the incidence of horizontally transmitted HBV infection in under-5-year-olds, leading to a significant reduction in the burden of the disease worldwide [63]. The WHO Africa regional office reports that all 47 member states have introduced routine childhood hepatitis B vaccination with the majority (94%) using the pentavalent vaccine (a combination vaccine for diphtheria, tetanus, hepatitis B, pertussis, and Haemophilus influenzae type B), administered in a 3-dose schedule at 6, 10, and 14 weeks of life [2]. However, various studies have demonstrated ongoing HBV infection in children despite the receipt of all 3 doses [4,64,65,66,67], suggesting susceptibility to infection from exposure early on at birth [19]. It is therefore clear that the current strategy is insufficient at interrupting HBV MTCT, which carries a higher likelihood of life-long infection and fatal outcomes.

#### 3.2.1. Barriers to Adopting Universal Hepatitis B Birth-Dose Vaccination Programs

The growing evidence presented so far argues for a substantial risk of HBV MTCT in Africa. The 2017 Global Hepatitis Report emphasizes the fact that hepatitis B birth-dose vaccination remains the cornerstone of preventing vertical transmission [63] due to its cost-effectiveness and far-reaching lifesaving benefits [3,22,68]. Cost-effectiveness of hepatitis B birth-dose vaccination, in particular, has been researched widely across Africa and proven beneficial in countries within Western, East, Southern, and Central Africa [3,22,68,69,70,71,72,73]. Despite this, only a third of the member states in the region have adopted the vaccine as part of their national immunization programs, having already missed interim targets set by the WHO Africa regional office of 25 countries adopting the vaccine by 2020 [28].

Key barriers to the adoption of hepatitis B birth-dose vaccination in countries within the region are cited in the 2016 WHO African regional situational report [48]. As reported by member states, these include (i) lack of financial support from Gavi, the Vaccine Alliance (10 countries), (ii) the need for further evidence on the burden of chronic HBV infection and the risk of perinatal transmission in Africa (6 countries), (iii) insufficient cold-chain storage (3 countries), (iv) a high proportion of home births (2 countries), and (v) a lack of trained health care workers (HCWs) in attending to birth or conducting post-natal visits (2 countries) [48]. In a PubMed literature review, Dionne-Odom et al. categorized four barriers to improved performance of hepatitis B birth-dose vaccination in the region [1]. These included (i) limited awareness of HBV prevalence and preventative measures, (ii) vaccine availability, (iii) out of facility deliveries, and (iv) cold-chain storage requirements [1]. Tamandjou Tchuem et al. further cite a lack of political willingness, poor service delivery arrangements, and inadequate cold-chain systems as reasons for the delay in introducing universal hepatitis B birth-dose vaccination in Africa [47]. These findings suggest the need for further high-level political commitment and system-wide approaches to mitigate these barriers and strengthen the prevention of HBV MTCT in Africa [47].

#### 3.2.2. Challenges Faced by Established Hepatitis B Birth-Dose Vaccination Programs

**Figure 4:**
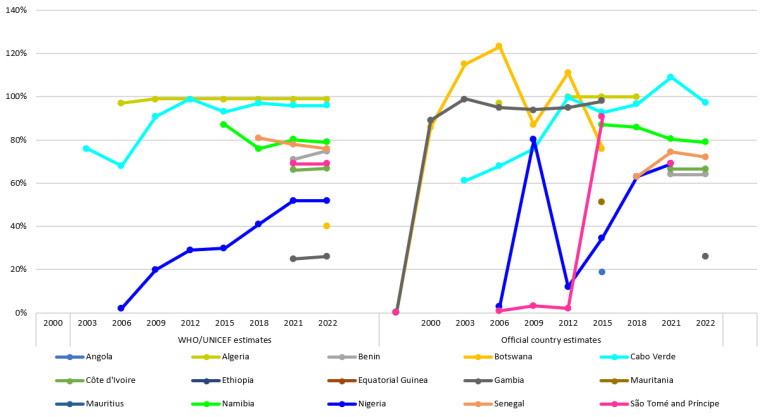
WHO/UNICEF (**left**) and official country (**right**) estimates of hepatitis B birth-dose vaccination coverage (2000–2022) [21,62].

In countries that have universal hepatitis B birth-dose vaccination programs in place, coverage estimates consistently show suboptimal and disparate uptake of the hepatitis B birth-dose vaccine (Figure 4). For example, in 2022, WHO/UNICEF estimates indicated that the coverage of the hepatitis B birth-dose vaccine ranged from 26% in The Gambia and 52% in Nigeria to 99% in Algeria [21,62]. Worth noting are the inconsistencies between the WHO/UNICEF and the official country estimates. This underscores the need for accurate and reliable coverage estimates to guide the strengthening of program performance. These disparate coverage rates may not provide comprehensive information on the timeliness of vaccination. In addition, they suggest persistent programmatic challenges in those countries reporting suboptimal coverage rates. Several studies have investigated the programmatic barriers to optimal uptake of the hepatitis B birth-dose vaccine in Africa, using both quantitative and qualitative research approaches. Some of the barriers reported across the literature include the lack of relevant policies at both national and health-facility levels [73,74], lack of funding or out-of-pocket payment requirements [75], poor monitoring and evaluation systems [73,74], lack of integration with the maternal and child health package [14,74], lack of awareness about HBV infection and hepatitis B birth-dose vaccination among pregnant women [75,76], geographical inaccessibility of immunization clinics [75,77], inaccessibility due to allotted vaccination days [74,75], frequent stockouts [75,78], home births [79,80], lack of outreach services [74], mistrust of HCWs handling newborns [76], birth doses administered on discharge only [74], poor knowledge of contraindications among HCWs, and absence of delineated staff [74]. In 2017, Breakwell et al. published a MEDLINE literature review citing (i) timely administration, (ii) the high prevalence of home births, (iii) the lack of services available to reach infants born at home, and (iv) unreliable vaccine supplies as challenges limiting improved adoption and coverage of the hepatitis B birth-dose vaccination program [2]. Moreover, a WHO systematic review on the global compliance with timely hepatitis B birth-dose vaccination reported a paucity of research evidence from the WHO Africa region compared to other regions, with only four publications representing four African countries meeting the inclusion criteria for that review [48]. Of the four, only one publication described programmatic barriers, which suggested that living in rural areas was the most weighted risk factor limiting access to the hepatitis B birth-dose vaccine post-delivery [48,77].

Limited access consistently emerges as a common thread across these reported barriers, whether it be due to the inability of national governments to secure necessary, sustainable financing mechanisms to procure hepatitis B birth-dose vaccines, limited access to information on birth-dose vaccination, geographical access barriers including limited access to immunization clinics, or, as frequently mentioned, poor access to the birth-dose vaccine for home births. In relation to the challenges associated with home births, expert opinions have reiterated the importance of encouraging institutional delivery, as the coverage of hepatitis B birth-dose vaccination is correlated with the rate of skilled birth attendance and institutional delivery, globally [81]. In a previous systematic review addressing Nigeria’s position on vertical transmission of hepatitis B, the rate of institutional delivery was reported to be only 35% and of those attending antenatal care, 58% [6]. Consequently, hepatitis B birth-dose vaccine coverage in Nigeria remains suboptimal (52%) despite integration with the national immunization schedule [80,82]. While calls for improved institutional delivery are valid [1,2,81], it remains important that national health systems become more responsive to cultural contexts where home births and isolation periods post-birth are concerned [2,76,77]. This may require tailored approaches to expanding the reach of the hepatitis B birth-dose vaccine within the African setting. This may include conducting community outreach and linkage to vaccination services and increasing community awareness through maternal education on HBV and hepatitis B birth-dose vaccination [2,6,77]. At the governance and policy level, there is a need to strengthen political commitment to and prioritization of global HBV elimination targets [1,47,81,83].

#### 3.2.3. Poor Adherence to Timely Hepatitis B Birth-Dose Vaccination

A priority concern facing hepatitis B birth-dose vaccination programs in Africa is the poor adherence to the recommended time of administration [2,84]. The Strategic Advisory Group of Experts on Immunization recommend administering the hepatitis B birth-dose within the first 24 h of life, although it remains effective against perinatal infection if administered within 7 days [2], and still beneficial in preventing early horizontal transmission if administered after 7 days [24]. Yet national policies in the region recommend a range of what is considered acceptable timely administration of hepatitis B birth-dose vaccination, from 24 h to as long as 2 weeks in countries like Namibia [2,74]. A study in Senegal found that only 54.5% of infants were vaccinated within 24 h and, in total, 58% within 7 days [79]. The situation is even more concerning in São Tomé and Príncipe where only 1.1% of infants have been reported to receive their hepatitis B birth-dose vaccination within 24 h of life [85]. In The Gambia, a review of 10 years of coverage data in a district found that only 1% of infants were vaccinated with hepatitis B birth-dose within 24 h, 5% within 7 days, and 58% within 28 days of life [2,77]. Similarly, in Nigeria, among children admitted to an emergency room, the mean age at hepatitis B birth-dose vaccination was 28 days [2]. Delayed uptake of the hepatitis B birth-dose vaccine could lead to the program being judged as ineffective at interrupting MTCT as neonates who do not receive the vaccine on time will have increased susceptibility to HBV infection. Furthermore, this inconsistency in adherence to the timing may negatively impact the potential for greater and effective uptake and adoption of hepatitis B birth-dose vaccination in other parts of the region.

Observations on other birth-dose vaccines in the region could offer further insights into the performance of programs with the same overall specification. In Africa, coverage of all three recommended birth-dose vaccines, namely, BCG, OPV, and hepatitis B birth-dose vaccines, were estimated by the WHO/UNICEF at 75%, 70%, and 17% in 2021, respectively [21]. While coverage data clearly shows poorer uptake of the hepatitis B birth-dose vaccine compared to BCG and OPV, it does not provide an indication of the timeliness of uptake of these vaccines. A systematic review by Bassoum et al. sought to investigate the timeliness of birth-dose vaccinations in sub-Saharan Africa and found that most neonates received their birth-dose vaccines within the first month of life, with coverage at this timepoint as follows: BCG at 71.7%; OPV at 76.1%; and hepatitis B birth-dose at 60.8% [61]. In comparison, coverage rates declined on day 7 (BCG at 48.7%, OPV at 53.8%, and hepatitis B birth-dose at 21.5%) and on days 0–1 (BCG at 14.2% and hepatitis B birth-dose at 1.3%) [61]. Coverage of OPV for the days 0–1 time point was not recorded by any of the included studies [61]. Overall, it is evident that within the Africa region, hepatitis B birth-dose vaccination programs achieve the lowest coverage rates and timeliness [61]. It is possible that the increased coverage and timeliness of BCG and OPV birth-dose vaccines can be attributed to their near-universal adoption (both vaccines have been included in the national immunization programs of 45 and 39 member states, respectively) and longstanding programs within Africa [61]. In comparison, universal hepatitis B birth-dose vaccination is only available in a limited number (15) of member states [61]. Nevertheless, greater efforts are required to ensure timely administration of the hepatitis B birth-dose vaccine as the combination of low vaccine coverage and non-adherence to the recommendations of administration may hamper efforts to achieve viral hepatitis elimination as a public health threat.

### 3.3. Health Systems’ Capacity to Support Hepatitis B Birth-Dose Vaccination Programs in the WHO Africa Region

Global agencies have recognized well-functioning health systems as crucial to the optimal performance of so-called vertical health programs and in attaining global health targets [32,86,87]. Despite effective and affordable interventions as well as access to international donor funding, fragile and fragmented health systems are often incapable of delivering key health services of adequate volume and quality [31,32,87]. Identifying programmatic barriers, though essential, is likely only an indication of the weaknesses that exist across the broader health system.

The country context has a considerable influence on the capabilities of the health system to effectively support the performance of health programs. Accordingly, barriers to the adoption and optimal performance of universal hepatitis B birth-dose vaccination programs in the region may be unique to the health systems that deliver them. For example, it has been previously suggested that countries with prolonged civil unrest and unstable governance, as observed previously in countries like Uganda and South Sudan, experience negative implications in the functioning of their health programs including hepatitis B birth-dose vaccination [11]. Furthermore, while countries like Nigeria contribute the largest amount of research on HBV MTCT in the region, the evidence generated has so far not translated into optimal coverage rates of the hepatitis B birth-dose vaccine [2,21]. Similarly, The Gambia, with more than 10 years of access to universal hepatitis B birth-dose vaccination, continues to experience inconsistencies with coverage rates [2,21]. This may point to the influence of contextual health-systems issues in these settings and may call for a “deeper understanding of the linkages, relationships, interactions and behaviors among elements that make up the entire system” [34]. Such an approach is better referred to as ‘systems thinking’ [34] and, as health systems are inherently complex [35], a more ‘complex systems thinking’ approach could be adopted. This could allow for the anticipation of system-wide effects as well as undesired synergies, which can then better inform mitigation strategies [34]. In doing so, system-level interventions can be modified, and more comprehensive evaluations designed for better monitoring of effects [34,87].

#### 3.3.1. Conceptual Models for the Assessment of Health Systems’ Capacity

According to the WHO, a health system encompasses “all organizations, people and actions whose primary intent it is to promote, restore and maintain health” [88]. This definition extends beyond just the delivery of health services and includes the acknowledgment of a complex people-centered system requiring the exploration and collaboration of multiple sectors for the attainment of health [88]. To promote a common understanding of health systems, the WHO provides a framework consisting of six dimensions (service delivery; health workforce; information; medical products, vaccines, and technologies; financing; and leadership and governance) referred to as building blocks [31,88]. Though several conceptual frameworks have attempted to capture the elements of health system performance in its entirety [86,88,89,90], the WHO health systems framework has become the most quoted framework in recent works [31].

Even though the WHO framework is effective in clarifying essential functions of the health system [88], it has been criticized for depicting the six dimensions in silos [31]. Realistically, it is the interdependence between the dimensions that is recognized as paramount for addressing programmatic challenges [88]. The interpretation of this model by de Savigny and Adam better explores the interaction between the dimensions in a non-linear relationship centered around the people within the health system [34]. The multiple relationships and interactions between the dimensions therefore result in the creation of a system [34]. Van Olmen et al. further emphasize the broader context in which a health system is embedded, the influence of its principles and values, and includes the population as part of the system [91]. Taken together, these conceptual models [34,88,91] emphasize the fact that health systems are complex systems [34]. Furthermore, complex systems are “dynamic, with interacting components—at various geographical levels—that lead to adaptation and emergence of new dynamics” [31].

#### 3.3.2. Complexity as a Characteristic of Hepatitis B Birth-Dose Vaccination Programs

Though it is established that not all interventions will benefit from a systems-thinking approach, complex interventions are likely to have profound effects across the system, and more so in weak health systems [34]. Among all three birth-dose vaccines endorsed by the WHO (BCG, OPV, hepatitis B birth-dose), universal hepatitis B birth-dose vaccination programs in Africa have performed the poorest, a fact likely attributable to the complexity of the intervention found in the limited degree of flexibility afforded in its requirement of timely administration within 24 h of life [38,61]. This has raised concerns on task shifting, cold storage, data capturing, and policy change [14,73,74], proving the complexity of the intervention across most, if not all, the health-systems building blocks. Characteristics describing intervention complexity have been conceptualized by many [34,37,39] and collated in a comprehensive list in Petticrew et al., assisting in the identification of sources of complexity [38]. With this in mind, it is likely that other sources of complexity associated with the intervention or the health system could provide further explanations for the underperformance of hepatitis B birth-dose vaccination programs in this region. In seeking complex explanations, complex approaches should be considered [36]. Using the principles of complex systems thinking and applying a systems lens in assessing existing hepatitis B birth-dose vaccination programs could facilitate a richer understanding of the aforementioned barriers and inform interventions aimed at scaling up the delivery of the program.

#### 3.3.3. A Systems-Based Logic Model for Assessing Complexity within Hepatitis B Birth-Dose Vaccination Programs

**Figure 5:**
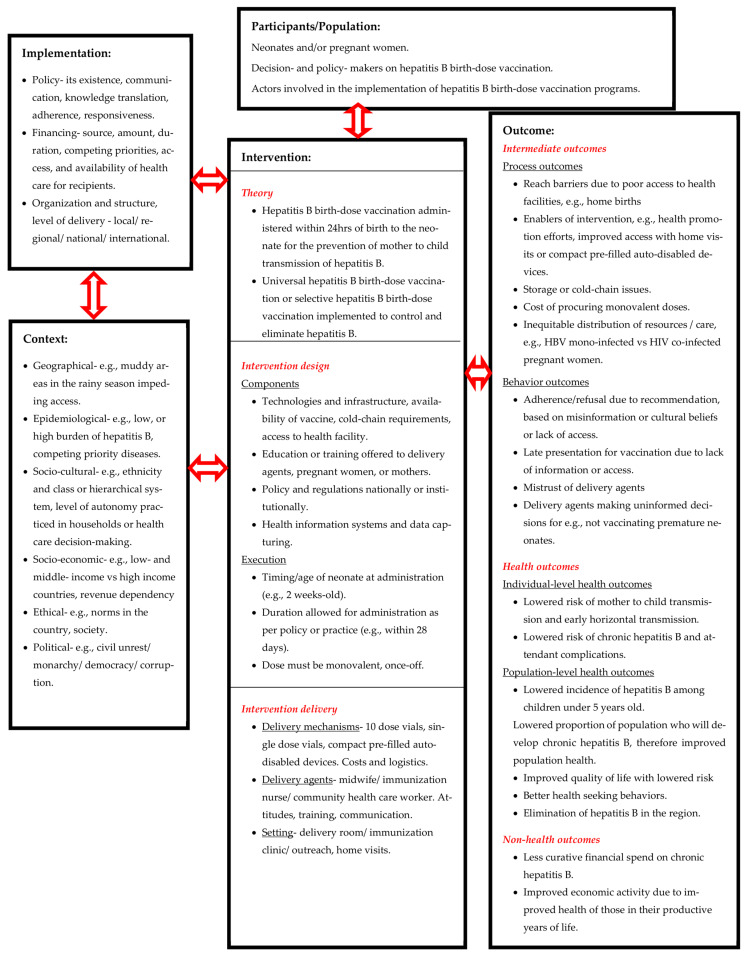
Systems-based logic model for understanding complexity of hepatitis B birth-dose vaccination programs [92].

We propose a systems-based logic model for understanding the role of complexity within hepatitis B birth-dose vaccination programs and the health systems that deliver them (Figure 5). This model is an adaptation of the template designed by Rohwer et al. for systematic reviews of complex interventions and builds on previous related frameworks [92,93,94]. It depicts the system in which interactions among the participants, the intervention (hepatitis B birth-dose vaccination), and the context take place [92]. The intervention is divided into theory, design, and delivery, with the expansion of these subcomponents into key aspects like process of execution, delivery mechanisms, and agents. Program implementation requires details on policy, financing, providers, organization, and structure, while context requires the description of the geographical, epidemiological, socio-cultural, socio-economic, ethical, legal, and political landscapes. Finally, outcomes are categorized into short, intermediate, and long term, with consideration for the nature of these outcomes [92]. Further description of the allocation of factors in the model can be found in Box 1. Application of this model in future evidence syntheses on hepatitis B birth-dose vaccination programs in the African region may provide a means of conceptualizing complexity and system-wide effects, making findings more accessible to a broad range of decision- and policy-makers [92].

Box 1 **Description of the allocation of factors to the systems-based logic model [92].**

Intervention—theory, design, and/or delivery elements

Intervention theory refers broadly to the description of implicit or explicit ideas on how an intervention works including the overall aims of the intervention.

Intervention design is descriptive of the “What?” of the intervention. The ‘execution’ provides a detailed prescription of intervention; elements of timing (when), duration (how long), dose (how much), and intensity (how often) are described.

Intervention delivery describes the “How?” (delivery mechanisms), “Who?” (delivery agents), and “Where? (setting)” of the intervention. Delivery agents, as individuals, form the basis of every organization and the potential for organizational change. Their knowledge, skills, motivation, and beliefs are vital for the success of intervention delivery.

Outcomes are categorized into those short, intermediate, and long term. Outcomes should include both desired/positive outcomes and potential undesired/negative outcomes.

Intermediate outcomes can be divided into process, behavior, and surrogate outcomes. Process outcomes are described quantitatively and/or qualitatively and include elements of participation, implementation fidelity, reach, experience of barriers, contamination by study or non-study interventions of the comparison group, and description of experiences of participants and intervention providers. Behavior outcomes include participant behaviors such as adherence or compliance, which are important for the success of the intervention but may extend to include intended or unintended behavioral outcomes.

Health outcomes include specific clinical outcomes, and may refer to broader outcomes, such as well-being and life expectancy.

Non-health outcomes include relevant societal impacts of the intervention.

The context and implementation section highlights the importance of a broader range of factors influencing the effectiveness of complex interventions.

## 4. Discussion

This scoping review maps the growing body of evidence on HBV MTCT in the WHO Africa region. The findings affirm the need for hepatitis B birth-dose vaccination programs in order to effectively interrupt HBV MTCT in Africa. Barriers to adoption and implementation of hepatitis B birth-dose vaccination programs underscore important gaps in broader health systems functioning. While previous reviews have applied a systems-thinking lens, both at a global [84] and regional [78] level, they fall short in the representation of research evidence from the WHO Africa region, rendering generalizability problematic.

In 2012, the WHO Immunization, Vaccines, and Biologicals Department summarized and appraised implementation evidence from 65 studies using the WHO health systems framework to categorize barriers and facilitators [84]. Recommendations emerging from that review ranged across all building blocks, some of which included encouraging governments to adopt a central policy on universal hepatitis B birth-dose vaccination as well as setting clear guidelines on the definition of ‘timely administration’, the meticulous tracking of pregnancies and birth by community HCWs to improve hepatitis B birth-dose vaccine coverage, and the usage of single-dose vials to discourage the practice of delaying hepatitis B birth-dose vaccination due to concerns with wastage when using multi-dose vials [84]. While the review sought to provide guidance for LMICs in implementing universal hepatitis B birth-dose vaccination programs, none of the included studies were from Africa or focused on the complexity of the intervention or system. More recently in 2022, Boisson et al. sought to identify literature focused on introducing hepatitis B birth-dose vaccination programs and barriers to uptake in sub-Saharan Africa [78]. The authors of this review summarized and categorized 39 relevant reports based on determinants of implementation at the policy, facility, and community levels [78]. Findings ranged from policy advocacy, facility supply and stockouts, to community HCW involvement, among other factors [78]. Again, while the authors sought to provide an overview of experiences from sub-Saharan Africa, the scoping extended to research conducted in the South-East Asian region and only included 13 publications from sub-Saharan Africa of the total 39 included studies [78]. Thus, while the evidence-based recommendations from these reviews are comprehensive and may meet the needs of policy-makers and immunization program managers in some LMICs, the findings may not always be generalizable to the African context.

Persistent barriers identified demonstrate the need to evade perceiving hepatitis B birth-dose vaccination programs as a simple intervention, especially when introduced and implemented in resource-constrained settings. With this perspective, a complex explanation should be sought in order to plan for successful vaccination programs. It is not only the complexity found in the timing requirements of the intervention but the way this interacts across the health system (service delivery arrangements, organizational capacity, political and cultural contexts, among others) that affects its success. Further exploration is needed to uncover what the sources of complexity are, and how we can use this information to mitigate persistent barriers encountered, as reiterated extensively in this review. Considering this, and the fact that previous reviews have not explored the systems complexity perspective, we propose the use of the systems-based logic model tailored to hepatitis B birth-dose vaccination programs. We further emphasize the need for research that is contextualized to the African setting in order for its application to African health systems.

Building on the findings of previous reviews, we contribute critical system-wide evidence underpinning the weak adoption and performance of hepatitis B birth-dose vaccination programs in this region [2]. Taken together, these findings underscore the urgency to scale-up universal hepatitis B birth-dose vaccination programs across Africa with careful consideration for underlying systems complexities. Key strengths of this scoping review include adherence to published methodological guidelines and the use of a systematic literature search across multiple electronic databases and relevant organizational websites. Despite this, our findings will have to be considered in light of some methodological limitations. While scoping reviews provide comprehensive and up-to-date information on a topic of focus, other evidence synthesis approaches like systematic reviews provide superior evidence with the lowest risk of bias. In addition, our review only included studies published in English, limiting the generalizability of the findings to Francophone and Lusophone countries from which potentially relevant research outputs may have been excluded.

## 5. Conclusions

The dearth of synthesized evidence needed to inform health system strengthening efforts to support hepatitis B birth-dose vaccination programs within Africa creates challenges in the adoption and effective performance of this pivotal intervention. Through well-funded health system strengthening efforts, it is possible to achieve optimal service delivery and, ultimately, improved health outcomes within the population. Such efforts will have to be informed by robust, context-specific evidence. Future research directions should include the exploration of contextualized complex systems approaches to scaling-up hepatitis B birth-dose vaccination programs within the African region.

## Data Availability

The complete search strategy and a summary of all extracted data is provided in the supplementary material.

## Supplementary Materials

The following supporting information can be downloaded at: https://www.mdpi.com/article/10.3390/tropicalmed8100474/s1, Table S1: Literature search strategy for the scoping review; Table S2: Summary of data extracted from the included literature.

## Appendix A

**Table A1:** Preferred Reporting Items for Systematic reviews and Meta-Analyses extension for Scoping Reviews (PRISMA-ScR) checklist [41].

**Section**	**Item**	**PRISMA-ScR checklist item**	**Page number**
**Title**			
Title	1	Identify the report as a scoping review.	1
**Abstract**			
Structured summary	2	Provide a structured summary that includes (as applicable): background, objectives, eligibility criteria, sources of evidence, charting methods, results, and conclusions that relate to the review questions and objectives.	1–21
**Introduction**			
Rationale	3	Describe the rationale for the review in the context of what is already known. Explain why the review questions/objectives lend themselves to a scoping review approach.	1–3
Objectives	4	Provide an explicit statement of the questions and objectives being addressed with reference to their key elements (e.g., population or participants, concepts, and context) or other relevant key elements used to conceptualize the review questions and/or objectives.	2–3
**Methods**			
Protocol and registration	5	Indicate whether a review protocol exists; state if and where it can be accessed (e.g., a Web address); and if available, provide registration information, including the registration number.	N/A
Eligibility criteria	6	Specify characteristics of the sources of evidence used as eligibility criteria (e.g., years considered, language, and publication status), and provide a rationale.	3
Information sources	7	Describe all information sources in the search (e.g., databases with dates of coverage and contact with authors to identify additional sources), as well as the date the most recent search was executed.	3
Search	8	Present the full electronic search strategy for at least 1 database, including any limits used, such that it could be repeated.	1–2 (Table S1)
Selection of sources of evidence	9	State the process for selecting sources of evidence (i.e., screening and eligibility) included in the scoping review.	3
Data charting process	10	Describe the methods of charting data from the included sources of evidence (e.g., calibrated forms or forms that have been tested by the team before their use, and whether data charting was done independently or in duplicate) and any processes for obtaining and confirming data from investigators.	3
Data items	11	List and define all variables for which data were sought and any assumptions and simplifications made.	3
Critical appraisal of individual sources of evidence	12	If done, provide a rationale for conducting a critical appraisal of included sources of evidence; describe the methods used and how this information was used in any data synthesis (if appropriate).	N/A
Synthesis of results	13	Describe the methods of handling and summarizing the data that were charted.	3
**Results**			
Selection of sources of evidence	14	Give numbers of sources of evidence screened, assessed for eligibility, and included in the review, with reasons for exclusions at each stage, ideally using a flow diagram.	5
Characteristics of sources of evidence	15	For each source of evidence, present characteristics for which data were charted and provide the citations.	7–8 &3–19 (Table S2)
Critical appraisal within sources of evidence	16	If done, present data on critical appraisal of included sources of evidence (see item 12).	N/A
Results of individual sources of evidence	17	For each included source of evidence, present the relevant data that were charted that relate to the review questions and objectives.	3–18
Synthesis of results	18	Summarize and/or present the charting results as they relate to the review questions and objectives.	3–18
**Discussion**			
Summary of evidence	19	Summarize the main results (including an overview of concepts, themes, and types of evidence available), link to the review questions and objectives, and consider the relevance to key groups.	20–21
Limitations	20	Discuss the limitations of the scoping review process.	21
Conclusions	21	Provide a general interpretation of the results with respect to the review questions and objectives, as well as potential implications and/or next steps.	21
**Funding**			
Funding	22	Describe sources of funding for the included sources of evidence, as well as sources of funding for the scoping review. Describe the role of the funders of the scoping review.	21
