# Supplementary material for: Weak Adoption and Performance of Hepatitis B Birth-Dose Vaccination Programs in Africa: Time to Consider Systems Complexity?—A Scoping Review
Source: Trop Med Infect Dis. 2023 Oct 16;8(10):474. doi: 10.3390/tropicalmed8100474 (PMC10611266; doi:10.3390/tropicalmed8100474)
Supplement: Supplementary file 1 [file tropicalmed-08-00474-s001.zip › tropicalmed-2592808-supplementary.pdf]

Table S1. Literature search strategy for the scoping review

Database	Type of search terms	Search Strategy	Results
**PubMed**<br><br>**(incl. MEDLINE)**	MeSH[1]<br><br>Keywords<br><br><br><br>LMIC filter[2]	Hepatitis B OR Hepatitis B Vaccines [Mesh]<br><br>OR Hepatitis B OR Hep B OR HBV OR Hepatitis B vaccine [keywords] AND Birth dose OR birth-dose OR BD or birth dose vaccine OR birth-dose vaccine OR BD vaccine [keywords]<br><br>AND Deprived Countries OR Deprived Population OR Deprived Populations OR Developing Countries OR Developing Country OR Developing Economies OR Developing Economy OR Developing Nation OR Developing Nations OR Developing Population OR Developing Populations OR Developing World OR LAMI Countries OR LAMI Country OR Less Developed Countries OR Less Developed Country OR Less Developed Economies OR Less Developed Nation OR Less Developed Nations OR Less Developed World OR Lesser Developed Countries OR Lesser Developed Nations OR LMIC OR LMICS OR Low GDP OR Low GNP OR Low Gross Domestic OR Low Gross National OR Low Income OR Lower GDP OR lower gross domestic OR Lower Income OR Middle Income OR Poor Countries OR Poor Country OR Poor Economies OR Poor Economy OR Poor Nation OR Poor Nations OR Poor Population OR Poor Populations OR poor world OR Poorer Countries OR Poorer Economies OR Poorer Economy OR Poorer Nations OR Poorer Population OR Poorer Populations OR Third World OR Transitional Countries OR Transitional Country OR Transitional Economies OR Transitional Economy OR Under Developed Countries OR Under Developed Country OR under developed nations OR Under Developed World OR Under Served Population OR Under Served Populations OR Underdeveloped Countries OR Underdeveloped Country OR underdeveloped economies OR underdeveloped nations OR underdeveloped population OR Underdeveloped World OR Underserved Countries OR Underserved Nations OR Underserved Population OR Underserved Populations OR Afghanistan OR Albania OR Algeria OR American Samoa OR Angola OR Armenia OR Azerbaijan OR Bangladesh OR Belarus OR Byelarus OR Belorussia OR Belize OR Benin OR Bhutan OR Bolivia OR Bosnia OR Botswana OR Brazil OR Bulgaria OR Burma OR Burkina Faso OR Burundi OR Cabo Verde OR Cape Verde OR Cambodia OR Cameroon OR Central African Republic OR Chad OR China OR Colombia OR Comoros OR Comores OR Comoro OR Congo OR Costa Rica OR Côte d'Ivoire OR Cuba OR Djibouti OR Dominica OR Dominican Republic OR Ecuador OR Egypt OR El Salvador OR Equatorial Guinea OR Eritrea OR Ethiopia OR Fiji OR Gabon OR Gambia OR Gaza OR Georgia OR Georgia Republic OR Ghana OR Grenada OR Grenadines OR Guatemala OR Guinea OR Guinea- Bissau OR Guyana OR Haiti OR Herzegovina OR Hercegovina OR Honduras OR India OR Indonesia OR Iran OR Iraq OR Ivory Coast OR Jamaica OR Jordan OR Kazakhstan OR Kenya OR Kiribati OR Democratic People's Republic of Korea OR Kosovo OR Kyrgyz OR Kirghizia OR Kirghiz OR Kyrgyzstan OR Lao PDR OR Laos OR Lebanon OR Lesotho OR Liberia OR Libya OR Macedonia OR Madagascar OR Malawi OR Malay OR Malaya OR Malaysia OR Maldives OR Mali OR Marshall Islands OR Mauritania OR Mauritius OR Mexico OR Micronesia OR Moldova OR Mongolia OR Montenegro OR Morocco OR Mozambique OR Myanmar OR Namibia OR Nepal OR Nicaragua OR Niger OR Nigeria OR Pakistan OR Palau	329

[1]MeSH: Medical Subject Headings thesaurus is a controlled and hierarchically organized vocabulary produced by the National Library of Medicine. It is useful when searching biomedical and health related information (http://www.nlm.nih.gov).
[2]LMIC filter: database specific LMIC filters have been compiled by the Health Sciences Faculty library, University of Cape Town (UCT) and available from their website (https://www.medical.lib.uct.ac.za/).

		OR Papua New Guinea OR Paraguay OR Peru OR Philippines OR Principe OR Romania OR Ruanda OR Rwanda OR Samoa OR Sao Tome OR Senegal OR Serbia OR Sierra Leone OR Solomon Islands OR Somalia OR South Africa OR South Sudan OR Sri Lanka OR St Lucia OR St Vincent OR Sudan OR Surinam OR Suriname OR Swaziland OR Syria OR Syrian Arab Republic OR Tajikistan OR Tadzhikistan OR Tajikistan OR Tadzhik OR Tanzania OR Thailand OR Timor OR Togo OR Tonga OR Tunisia OR Turkey OR Turkmen OR Turkmenistan OR Tuvalu OR Uganda OR Ukraine OR Uzbek OR Uzbekistan OR Vanuatu OR Venezuela OR Vietnam OR West Bank OR Yemen OR Zambia OR Zimbabwe	
**EBSCO host**<br>**(Databases: Academic Search Premier; Africa-Wide Information; CINAHL; Health Source: Nursing/Academic Edition; APA PsycInfo)**	Keywords<br>LMIC filter	Hepatitis B OR Hep B OR HBV OR Hepatitis B vaccine [keywords] AND Birth dose OR birth-dose OR BD or birth dose vaccine OR birth-dose vaccine OR BD vaccine [keywords]<br>(As per footnote [2])	248
**Scopus**	Keywords<br>Keywords<br>LMIC filter	ALL (Hepatitis B OR Hep B OR HBV OR Hepatitis B vaccine) AND<br>ALL (Birth dose OR birth-dose OR BD or birth dose vaccine OR birth-dose vaccine OR BD vaccine) AND<br>TITLE-ABS-KEY (as per footnote [2])	247
**Web of Science (excl. MEDLINE)**	Keywords<br>Keywords<br>LMIC filter	Hepatitis B OR Hep B OR HBV OR Hepatitis B vaccine [keywords] AND<br>Birth dose OR birth-dose OR BD or birth dose vaccine OR birth-dose vaccine OR BD vaccine [keywords]<br>As per footnote [2]	26
**Google Scholar**	Keywords<br>Keywords<br>LMIC filter	Hepatitis B birth dose (all in title) AND<br>Vaccine OR vaccination OR vaccinated (at least one in title) AND<br>As per footnote [2] (at least one in title)	141

Table S2. Summary of data extracted from included literature

No.	Author, Year	Country	Type of study	Title	Key findings
1.	Breakwell et al., 2017 [2]	WHO AFRO	Literature review (Medline review)<br><br>Data review of African region (relevant data on coverage, annual births, etc.)	The status of hepatitis B control in the African region	These findings reinforce the need to improve hepatitis B infant vaccination (Hep B3) coverage and that some countries might need to consider introducing a hepatitis B birth-dose vaccination to achieve the regional hepatitis B control goal.<br>Ultimately, all countries will need the hepatitis B birth-dose vaccine to make further progress to eliminate HBV MTCT by 2030. Strong political commitment, clear policy recommendations, and training of staff on vaccination administration and documentation are required.
2.	Shimakawa et al., 2014 [44]	The Gambia	Case-control study	The association between maternal hepatitis B e antigen status, as a proxy for perinatal transmission, and the risk of hepatitis B e antigenaemia in Gambian children	Having an HBeAg-positive mother was a risk factor for HBeAg positivity in children carrying HBsAg (adjusted OR 4.5, 95% CI: 1.0-19.5, $p = 0.04$), whilst the number of HBeAg-positive older siblings was not. Maternal HBeAg was associated with positive HBeAg in children with chronic HBV infection. This suggests that interrupting mother-to-infant transmission in sub-Saharan Africa might help reduce the burden of liver disease. A timely dose of HBV vaccine within 24 hours of birth, as recommended by WHO, should be implemented in sub-Saharan Africa.
3.	Apiung et al., 2017 [65]	Ghana	Cross-sectional study	Hepatitis B virus surface antigen and antibody markers in children at a major paediatric hospital after the pentavalent DTP-HBV-Hib vaccination	Residual possibility of Hepatitis B infection despite infant vaccination series. Neonatal vaccination with Hepatitis B birth-dose should be considered.
4.	Rashid et al., 2014 [12]	Tanzania	Cross-sectional study	Seroprevalence of hepatitis B virus infection among antenatal clinic attendees at a tertiary hospital in Dar es Salaam, Tanzania	Seroprevalence of HBsAg among women attending antenatal care at Muhimbili National Hospital is moderate. It is recommended to introduce routine antenatal screening for HBV and "at birth dose" vaccination to newborn babies of mothers found to be HBsAg positive.
5.	Kirbak et al., 2017 [11]	South Sudan	Cross-sectional study	Sero-prevalence for Hepatitis B virus among pregnant women attending antenatal clinic in Juba Teaching	Prevalence of Hepatitis B surface antigen among pregnant women attending antenatal care in Juba was 11% (31 out of the 280 samples). In Juba county 70% of babies would be at risk of infection, if a birth-dose of Hepatitis B is not provided. Recommendation of the introduction of Hepatitis B Vaccine birth dose into routine infants' vaccination series to eliminate this risk.

				Hospital, Republic of South Sudan	
6.	Cheung, 2017 [85]	São Tomé and Príncipe	Cross-sectional study<br><br>Dissertation (Uppsala University publications)	Proportion of children born to infected mothers at risk of contracting Hepatitis B, and associated risk factors for inadequate Hepatitis B Timely Birth Dose vaccination: Analysis of the São Tomé and Príncipe Demographic Health Survey Program data, 2008-2009	Vaccination coverage is high but quality, namely timing, is poor. Only 1.1% of those receiving their first dose at birth had timely administration. Associated factors to timely birth-dose linked to socioeconomic factors. Further qualitative research should explore this. Policy needs to address the quality of hepatitis B birth-dose vaccinations.
7.	Howell et al., 2014 [5]	sub-Saharan Africa (SSA)	Literature review	Prevention of materno-foetal transmission of hepatitis B in sub-Saharan Africa: the evidence, current practice and future challenges	HBV vaccine coverage remains low, and HBV birth-dose vaccination has not been implemented. HBV transmission from mother to child in the early perinatal period therefore remains a significant contributor to the burden of HBV-related disease in sub-Saharan Africa.
8.	Tamandjou Tchuem et al., 2021 [3]	Namibia	Cost-effectiveness analysis	Prevention of hepatitis B mother-to-child transmission in Namibia: A cost-effectiveness analysis	At the Namibian cost/DALY averted threshold of US$3 142, the (1) BD vaccination + targeted HBIG, and (2) maternal antiviral prophylaxis with sequential HBeAg testing interventions were cost-effective.<br>The analysis showed that elimination of HBV MTCT is achievable using maternal antiviral prophylaxis with active and passive immunization.
9.	Sone et al., 2017 [7]	Cameroon	Prospective Cohort and Cross-sectional study	Prevalence and identification of serum markers associated with vertical transmission of hepatitis B in Pregnant women in Yaounde, Cameroon	HBeAg and increase in liver transaminases were serum markers associated with the vertical transmission of HBV while HBeAb and anti-HIV therapy were protective markers.<br>There is need to systematically screen all pregnant women for hepatitis B, follow up those that are positive, and administer a dose of gammaglobulin anti-HBs to their children to reduce the risks of chronic hepatitis and hepatocellular carcinoma.
10.	Chotun et al., 2017 [15]	South Africa	Observational prospective cohort study	Point-of-care screening for hepatitis B virus infection in pregnant women at an antenatal clinic: A South African experience	Six HBV-exposed infants received the HBV vaccine within 24 hours of birth, of whom two were lost to follow-up and four had undetectable levels of HBV DNA when tested at the two time points.<br>HBV screening using POC testing fulfilled the criteria considered necessary for implementation. It has acceptable performance, is inexpensive, reliable, and was well accepted by the study participants. Screening pregnant women

					as part of the HBV MTCT prevention strategy is therefore feasible in a South African clinical setting.
11.	Metodi et al., 2010 [66]	Tanzania	Cross-sectional study	Immunity to hepatitis B vaccine in Tanzanian under-5 children	Five children (1.7%) were positive for HBsAg, suggesting possible vertical transmission. More than two-thirds of children under 5 years had protective anti-HBs levels. A change in the hepatitis B immunization schedule to include a dose immediately after birth should improve immunity.
12.	Guingané et al., 2020 [13]	Burkina Faso	Prospective cohort study	Identifying gaps across the cascade of care for the prevention of HBV mother-to-child transmission in Burkina Faso: Findings from the real world	In this study, of 5200 pregnant women consulting for the antenatal visit, 2261 (43.5%) were proposed pre-test counselling and HBsAg screening and 2220 (98.2%) have agreed to screening. Among 1580 (71.2%) women that came back for the post-counselling interview, 75 were positive for HBsAg (4.8%), 73 (97.3% of the women provided HBsAg result) consented to medical consultation with hepatogastroenterologists and 53 (72.6%); performed the HBV DNA testing. Forty-seven out of 60 (78.3%; 65.8-87.9) children born alive were immunized for HBV within 24 hours of life. Retention in care was associated with the level of education of the infant's father, secondary school or higher was associated with a better retention in care of the women (OR: 6.6; P=0.03). The study shows large gaps in HBV PMTCT.
13.	Tamandjou Tchuem et al., 2017 [47]	sub-Saharan Africa	Commentary	Is hepatitis B birth dose vaccine needed in Africa?	Africa needs the HBV birth dose vaccine if we are to achieve the goal of eliminating HBV MTCT. Screening pregnant women for HBV and treating those with high viral loads would further reduce the risk of vertical transmission. The combined use of these practices would serve to effectively break the HBV transmission cycle.
14.	Bayo et al., 2014 [10]	Uganda	Cross-sectional study	High prevalence of hepatitis B virus infection among pregnant women attending antenatal care: A cross-sectional study in two hospitals in northern Uganda	One in eight pregnant women attending antenatal care in the two study hospitals has evidence of hepatitis B infection. A significant number of these mothers are HBeAg positive and may be at increased risk of transmitting hepatitis B infection to their unborn babies. We suggest that all pregnant women attending antenatal care be tested for HBV infection; exposed babies need to receive HBV vaccines at birth.
15.	Chotun et al, 2015 [4]	South Africa	Cross-sectional study	Hepatitis B virus infection in HIV-exposed infants in the Western Cape, South Africa	This study demonstrates HBV infection in HIV-exposed infants despite HB vaccination from 6 weeks of age. A more strategic approach is needed to prevent mother to child transmission of HBV, including screening of pregnant women, HBV-targeted antiviral therapy and HB birth dose vaccine.
16.	Umare et al., 2016 [9]	Ethiopia	Cross-sectional study	Hepatitis B Virus infections and associated factors among pregnant women attending antenatal care clinic at	This study determined that the prevalence of HBV infection among pregnant women was 6.9%, implying that it is a high-intermediate endemic area, and reiterating that it is an important public health issue needing to be addressed. Furthermore, all pregnant women should be screened for HBV, treated if necessary to reduce their viral loads and their children vaccinated

				Deder Hospital, eastern Ethiopia	at birth with the single-dose hepatitis B vaccine to break the cycle of mother-to-child transmission.
17.	Bittaye et al., 2019 [8]	The Gambia	Cross-sectional study	Hepatitis B virus sero-prevalence amongst pregnant women in the Gambia	The prevalence of hepatitis B infection is very high among pregnant women at Edward Francis Small Teaching Hospital in the high endemic zone, that is more than 8%. However the prevalence rate is lower than the national average of 15%. The prevalence is of moderate endemicity among the women who likely received vaccination during childhood. More interventions during pregnancy need to be undertaken if more successes are to be registered.
18.	Sanou et al., 2018 [64]	Burkina Faso	Cross-sectional study	Hepatitis B vaccination in Burkina Faso: prevalence of HBsAg carriage and immune response in children in the western region	Of the 9 HBsAg positive children, 5 had HBsAg positive mothers. Despite good vaccination coverage (82.6%), a considerable proportion of vaccinated children remains unprotected from HBV infection. That emphasizes the need for further strengthening of the vaccination program through implementing the birth dose of HBV vaccine as recommended by the WHO.
19.	Dionne-Odom et al., 2018 [1]	sub-Saharan Africa	Literature review (PubMed review)	Elimination of vertical transmission of hepatitis B in Africa: A review of available tools and new opportunities	The most cost-effective intervention to reduce HBV infection rates in SSA is timely birth dose vaccination followed by completion of the 3-dose infant vaccine series. The identification and treatment of pregnant women with elevated HBV viral load to further reduce the risk of vertical transmission in SSA shows promise but efficacy and safety trials in Africa are lacking.
20.	Seremba et al., 2017 [49]	Uganda	Prospective cohort study	Early childhood transmission of hepatitis B prior to the first hepatitis B vaccine dose is rare among babies born to HIV-infected and non-HIV infected mothers in Gulu, Uganda	Vertical transmission does not seem to contribute substantially to the high HBV endemicity in northern Uganda. Among the HBV positive mothers, none of their babies tested positive. This finding is likely confounded by the use antiretrovirals by HIV co-infected mothers. The current practice of administering the first HBV vaccine to babies in Uganda at six weeks of age may be adequate in control of HBV transmission.
21.	Sadoh et al., 2014 [6]	Nigeria	Review	Does Nigeria need the birth dose of the hepatitis B vaccine?	These data indicate that there is a significant potential for vertical transmission of HBV in Nigerian infants providing a compelling reason for the continued use of the birth dose of the HBV vaccine.
22.	Thompson et al., 2021 [14]	Democratic Republic of Congo (DRC)	Prospective cohort feasibility study	Arresting vertical transmission of hepatitis B virus (AVERT-HBV) in pregnant women and their neonates in the Democratic Republic of the Congo: a feasibility study	4016 women were approached and screened, 2.7% positive HBsAg.<br>Of 109, 91 (83%) were eligible. Eleven percent of 91 were high risk infection, 9/10 high risk were put on ART (1 refused), 5 achieved viral suppression. 68% infants received hepatitis B birth-dose vaccination and 77% of them were timely. No MTCT HBV observed.<br>The study procedures were considered highly acceptable (>80%) among mothers. Recommendation: Adding HBV screening and treatment of pregnant women and infant birth-dose vaccination to existing HIV

					prevention of mother-to-child transmission platforms is feasible in countries such as the DRC. Birth-dose vaccination against HBV infection integrated within the current EPI and HIV prevention of mother-to-child transmission program could accelerate progress toward HBV elimination in Africa.
23.	Diale et al., 2016 [23]	South Africa	Retrospective cohort study	Antenatal screening for hepatitis B virus in HIV-infected and uninfected pregnant women in the Tshwane district of South Africa	Significantly higher HBV prevalence in HIV infected as opposed to HIV uninfected women. HBeAg prevalence between the two groups indicates that both groups were at an increased risk of vertical transmission, demonstrating a need for antenatal screening for HBV. Since antenatal HBV screening is often not affordable in low-income countries, there is a high demand for a birth-dose of HBV vaccine for prevention of vertical transmission to the neonates. Recommendation: South Africa current HBV vaccination schedule to include a birth-dose of HBV vaccine to cater for neonates exposed to HBV during pregnancy, particularly those born to HIV uninfected and to HBV-HIV co-infected mothers.
24.	Gosset et al, 2021 [69]	Senegal	Micro-costing study (economic and financial costing)	The Costs of introducing the hepatitis B birth dose vaccine into the national immunization programme in Senegal (NéoVac Study)	Total economic costs were USD 143,364 in 2015, USD 759,406 in 2016 and USD 867,311 in 2017, while financial costs were USD 127,745, USD 82,519 and USD 29,853, respectively. The economic (financial) cost per vaccinated newborn was USD 2.10 (USD 0.30) in 2016 and USD 1.90 (USD 0.20) in 2017. This study confirms findings from two studies suggesting the cost-effectiveness of introducing hepatitis B birth-dose (HepB-BD) into sub-Saharan African EPI.
25.	Hagan et al, 2019 [73]	São Tomé and Príncipe (STP)	Cost-effectiveness analysis	Selective Hepatitis B birth-dose vaccination in São Tomé and Príncipe: A program assessment and cost-effectiveness study	STP lacked national or facility-specific written policies and procedures related to HepB-BD vaccination. Timely HepB-BD to eligible newborns was considered a high priority, although timeliness of HepB-BD was not monitored. Compared with the existing selective vaccination strategy, universal HepB-BD would result in a 19% decrease in chronic HBV infections per year at overall cost savings of approximately 44%. Expansion to universal newborn HepB-BD without maternal screening is feasible and could result in cost savings if actual implementation costs and effectiveness fall within the ranges modeled.
26.	Memirie et al., 2020 [71]	Ethiopia	Cost-effectiveness analysis: Markov/decision analytic model	Introduction of birth dose of hepatitis B virus vaccine to the immunization program in Ethiopia: an economic evaluation	In Ethiopia, where the prevalence of HBV among pregnant women is 5%, adding a birth dose of HBV vaccine would present an incremental cost-effectiveness ratio (ICER) of USD 110 per DALY averted. The estimated ICER compares very favorably with a willingness-to-pay level of 0.31 times gross domestic product per capita (about USD 240 in 2018) in Ethiopia. Introducing a birth dose of HBV vaccine in Ethiopia would likely be highly cost-effective.
27.	Gosset et al., 2021 [69]	Burkina Faso	Cost-effectiveness analysis:	Cost-effectiveness of adding a birth dose of hepatitis B vaccine in the	Despite uncertainty about the magnitude of HepB-BD effectiveness, this study provides evidence that adding HepB-BD to HepB3 would be cost-effective in Burkina Faso - a nation where HBV is highly endemic.

			Decision tree and Markov modelling	Dafra district of the Hauts-Bassins Region in Burkina Faso (NéoVac Study)	
28.	Anderson et al., 2018 [22]	SSA: focus on Cameroon	Cost-effectiveness analysis: Decision analytic modelling	A decision analytic model for prevention of hepatitis B virus infection in Sub-Saharan Africa using birth-dose vaccination	The study indicated the addition of a universal birth-dose HBV vaccine to the pentavalent vaccine prevented the greatest number of additional HBV infections and was the preferred strategy at a willingness to pay threshold of $150 per infection prevented when the maternal HBV seroprevelance was greater than 6%. Findings support WHO recommendations for universal birth-dose HBV vaccine administration irrespective of the maternal HBV status.
29.	Klingler et al., 2012 [68]	Mozambique	Cost-effectiveness analysis: Markov modelling	Cost-effectiveness analysis of an additional birth dose of Hepatitis B vaccine to prevent perinatal transmission in a medical setting in Mozambique	An incremental cost-effectiveness ratio (ICER) for the additional birth dose of 250.95 US$ per DALY averted. Assuming a willingness-to-pay threshold of 441 US$, which was the GDP per capita for Mozambique in 2008, the findings show the additional birth dose to be highly cost-effective. But more information on the parameters that render the birth dose cost-ineffective in sensitivity analysis is needed.
30.	Reardon et al., 2019 [72]	Djibouti<br>Algeria<br>Mauritania<br>(Refugee populations)	Cost-effectiveness analysis	Cost-effectiveness of birth-dose hepatitis B vaccination among refugee populations in the African region: a series of case studies	Based on the model, addition of HepB BD vaccination is very cost-effective among three sub-Saharan refugee populations, using relative life-years saved. This analysis shows the potential benefit of implementing HepB BD vaccination among other camp-based refugee populations as more African countries introduce national HepB BD policies.
31.	Immunization, Vaccines and Biologicals, 2020 [84]	Global	Scoping review	Practices to Improve Coverage of the Hepatitis B Birth Dose Vaccine	Results categorized in health system parameters of service delivery, health workforce, medical technologies, health information systems, financing, community concerns/lack of knowledge, leadership, and governance. Each category with a list of recommendations based on the current evidence. E.g. increasing skilled birth attendance, integration with maternal child health services.
32.	Okenwa et al., 2019 [75]	Nigeria	Cross-sectional survey	Maternal reasons for non-receipt of valid Hepatitis B birth dose among mother-infant pairs attending routine immunization clinics, South-east, Nigeria	Overall, 254 (73.8%) infants did not receive valid HepB-BD.<br>Major reasons for its non-receipt: vaccine not available at place of delivery: delivery did not take place on immunization day; lack of awareness on timing of valid HepB-BD; long distance from the health facility; fee payment for immunization. Of the 384 maternal recommendations: 143 (37.2%) emphasized female literacy, 87 (22.7%) indicated pre-positioning the vaccines at labor rooms to improve valid HepB-BD uptake. Lack of maternal awareness on timing of HepB-BD and poor integration of child delivery and immunization services. Recommendation: educating mothers on benefits of a timely HepB-BD and pre-positioning the vaccines at the labor rooms.

33.	Moturi et al., 2018 [74]	Botswana<br>The Gambia<br>Namibia<br>Nigeria<br>São Tomé and Príncipe	Cross-sectional descriptive study	Implementing a birth dose of hepatitis B vaccine in Africa: Findings from assessments in 5 countries	Barriers to timely HepB-BD included absence of standard operating procedures delineating staff responsible for HepB-BD, not integrating HepB-BD into essential newborn packages, administering HepB-BD at the point of maternal discharge from facilities, lack of daily vaccination services, sub-optimal staff knowledge about HepB-BD contraindications and age-limits, lack of outreach programs to reach babies born outside facilities, and reporting tools that did not allow for recording the timeliness of HepB-BD doses.
34.	Allison et al, 2017 [81]	Global	Ecological study	Hepatitis B vaccine birth dose coverage correlates worldwide with rates of institutional deliveries and skilled attendance at birth	HepB-BD, institutional delivery rates and skilled birth attendance rates were significantly correlated in the World Health Organization African, South-East Asia and Western Pacific Regions. Increasing IDR and SBA rates, training and supervising staff, increasing community awareness, and using HepB-BD outside the cold chain where needed would increase HepB-BD coverage.
35.	Périères et al, 2021 [79]	Senegal	Cross-sectional survey study	Hepatitis B vaccination in Senegalese children: Coverage, timeliness, and sociodemographic determinants of non-adherence to immunisation schedules	Of 241 children with available vaccination data, 71.5% had received Hep-BD. Of these, 54.5% had received it within 24hr of birth, while 58.2% received it within seven days of birth. Being born in 2016 (the year the BD was introduced in Senegal's EPI) (versus 2017–2018) was associated with non-adherence to the BD. Moreover, being born during the dry season and at home (versus in a healthcare facility) were associated with non-adherence to the BD schedule. Outreach vaccination activities must be strengthened to increase coverage in children born at home, those living far from healthcare posts, and those in agriculturally poorer households. In addition, vaccination timeliness should be considered when evaluating the effectiveness of the current HBV vaccination program in Senegal.
36.	Nankya-Mutyoba et al., 2021 [76]	Uganda	Qualitative study	Hepatitis B birth dose vaccination for newborns in Uganda: A qualitative inquiry on pregnant women's perceptions, barriers and preferences	Structured focus group discussion explored birth dose awareness, perceptions, barriers and preferences: None had known about birth-dose vaccination, older women knew about liver cancer but not HBV, liver Ca was identified as being fatal. Concerns of safety and trust of HCWs handling newborns. Most participants preferred birth-dose be given at delivery in the mother's presence, oral over injectable vaccines, receiving Hep-BD education at antenatal consults.
37.	World Health Organization, 2016 [48]	Global	Systematic Review<br><br>And<br><br>Survey directed at the WHO African and	Global compliance with Hepatitis B vaccine birth dose and factors related to timely schedule	Only 4 publications identified in Africa, 3 of them spoke to coverage and only one community survey. This publication described social and demographic factors related to timely birth dose in The Gambia: living in rural areas was the most important risk factor for not receiving a birth dose.

			South-east Asia regional offices		The regional offices provided the answers in consultation with the countries identifying the most common barriers in the region to introducing the Hep B-BD are:<br>•Funding for birth dose program<br>•The percentage of births that take place outside health facilities.<br>•Insufficient disease burden data<br>•Vaccine storage and access to cold chain<br>•Central policies and guidelines
38.	Maponga et al., 2019 [83]	South Africa	Review	Sustainable Development Goals for HBV elimination in South Africa: challenges, progress, and the road ahead	Poor funding, lack of advocacy and education, and the stigma associated with infection represent further barriers. Widespread deployment of the HBV vaccine in infancy has already markedly reduced pediatric infection, and birth dose vaccinations will further reduce the risk of vertical transmission. South Africa can capitalize on the laboratory and clinical infrastructure that already supports HIV diagnosis and treatment, by expansion of services to include HBV. HBV treatment and diagnostics are now on WHO lists of essential drugs and equipment. GAVI's vaccine plan for 2021-2025 includes consideration of funding for widespread HBV BD vaccination.
39.	Olakunde et al., 2021 [80]	Nigeria	Cross-sectional study	The coverage of hepatitis B birth dose vaccination in Nigeria: Does the place of delivery matter?	The association between HepB-BD vaccination and the place of delivery was assessed. About 53% of the children received the HepB-BD vaccine. The percentage of those who received the BD respectively in terms of place of delivery are approximately 77% in a public health facility, 83% in a private health facility and 33% at home. After controlling for child- and maternal-level factors, the odds of receiving HepB-BD vaccine were significantly lower in children delivered at a private health facility. Wealth index and region of residence were significantly associated with the receipt of HepB-BD vaccination in all three places of delivery. There is a need for private health sector engagement for immunization service delivery and innovative community-based interventions to reach the children delivered at home.
40.	Andersson et al., 2013 [19]	South Africa	Retrospective cross-sectional study	The epidemiology of hepatitis B virus infection in HIV-infected and HIV-uninfected pregnant women in the Western Cape, South Africa	Use of antenatal sentinel HIV and Syphilis Prevalence Survey in the Western Cape, South Africa. All HIV-infected women were age and race-matched to HIV-uninfected women. Study showed a trend toward loss of immune control of HBV in HIV-infected women with 3.4% of samples containing HBsAg, 18.9% contained HBeAg. In contrast, 2.9% of samples from HIV-uninfected women contained HBsAg and 17.1% of these HBeAg. HIV-HBV co-infected women exhibit a degree of immune escape. One in six HBV-infected pregnant women, irrespective of HIV status is HBeAg seropositive. HBV immunization of newborns in sub-Saharan Africa should be implemented.

41.	Jooste et al., 2016 [58]	South Africa	Prospective cohort study	Screening, characterization and prevention of Hepatitis B virus (HBV) co-infection in HIV-positive children in South Africa	HIV-positive children were screened and followed up. The screening campaign was successfully incorporated into routine out-patient care. Among 625 patients tested, five were found positive for HBsAg (0.8%), of whom three were Hepatitis B e-antigen positive. Two additional children initially tested HBsAg-positive but were negative on repeat testing. Further evaluation of the optimum timing of the first vaccine HBV vaccine dose is required; a vaccine dose at birth could reduce prevalence further.
42.	Sichone et al., 2019 [53]	Zambia	Cross-sectional study	Prevalence of hepatitis B Virus, HIV and HBV coinfection and associated factors in pregnant women attending antenatal care at the University Teaching Hospital, Lusaka, Zambia	Equal number of HIV-negative and positive pregnant women were recruited. Of the 316 study participants 11(3.5%) tested positive for HBsAg. No statistical difference between HIV negative and positive (3.8% and 3.2% respectively) in terms of coinfection although being on combined anti-retroviral therapy had a 91% reduced odds of co-infection with HBV. However, HIV antiretroviral treatment seems to have a protective effect on acquisition of HBV infection. All pregnant women should routinely be screened for HBV so that babies born to high-risk mothers can receive the birth doses of HBV vaccine and immunoglobulins to prevent transmission to newborns.
43.	Platt et al., 2020 [52]	Global	Systematic review	Prevalence and burden of HBV co-infection among people living with HIV: A global systematic review and meta-analysis	Globally, the prevalence of HIV-HBsAg co-infection is 7.6% (IQR 5.6%-12.1%) in PLHIV, or 2.7 million HIV-HBsAg co-infections (IQR 2.0-4.2). The greatest burden (69% of cases; 1.9 million) is in sub-Saharan Africa. Odds of HBsAg infection were 1.4 times higher among PLHIV compared to HIV-negative individuals. Therefore, a high global burden of HIV-HBsAg co-infection, especially in sub-Saharan Africa exists. Key prevention strategies include infant HBV vaccination, including a timely birth-dose. Findings also highlight the importance of targeting PLHIV, especially high-risk groups for testing, catch-up HBV vaccination and other preventative interventions. The global scale-up of antiretroviral therapy for PLHIV using a tenofovir-based regimen provides an opportunity to simultaneously treat those with HBV co-infection, and in pregnant women to also reduce mother-to-child transmission of HBV alongside HIV.
44.	Hipgrave et al., 2006 [59]	Global	Literature review	Improving birth dose coverage of hepatitis B vaccine	The reports reviewed show that the vaccines studied were safe and effective whether stored cold or outside-of-cold-chain (OCC). Field and laboratory data also verifies the retained potency of the vaccine after exposure to heat. The attachment of a highly stable variety of a vaccine vial monitor (measuring cumulative exposure to heat) on many HepB vaccines strongly supports policies allowing their storage OCC, when this will benefit birth dose coverage. We recommend that this strategy be introduced to improve birth-dose coverage, especially in rural and remote areas.

45.	Dadari et al., 2021 [60]	Africa	Scoping review	How the use of vaccines outside the cold chain or in controlled temperature chain contributes to improving immunization coverage in low- and middle-income countries (LMICs): A scoping review of the literature	The World Health Organization recommends the use of vaccines under a 'controlled temperature chain' (CTC), which is an innovative approach of keeping vaccines outside the recommended +2 to +8°C, under proper monitoring for a limited time before administration. Kolwaite et al and Breakwell et al investigated HepB BD. Kolwaite et al found 27% median increase in coverage. Breakwell et al observed increased coverage from 30% to 68% and 4% to 24% in facility and home births respectively. Breakwell did find wastage was high and shortages were common. Other vaccines, which are yet to be labeled CTC, such as Hepatitis B (HepB), remain potent and effective when stored at room temperatures.
46.	Ekra et al., 2008 [46]	Côte d'Ivoire	Non-randomized vaccine effectiveness trial	A non-randomized vaccine effectiveness trial of accelerated infant hepatitis B immunization schedules with a first dose at birth or age 6 weeks in Côte d'Ivoire	Strategy 1: 0,6,14-week HepB vaccination; Strategy 2: 6,10,14 (status quo). At age 9 months, 0.5% of infants in both cohorts were positive for HBsAg and all were born to HBeAg-positive women. Among infants of HBeAg-positive mothers, 9 of 24 (37.5%) in the birth cohort and 10 of 17 (58.8%) in the 6-week cohort were HBsAg. While both vaccine schedules prevented most cases of infant HBV transmission, both also had high failure rates among infants of HBeAg-positive mothers. African infants may benefit from a birth-dose, but additional studies are needed to verify this hypothesis.
47.	World Health Organization, 2016 [25]	Global	Organization publication	Global health sector strategy on viral hepatitis 2016-2021. Towards ending viral hepatitis	Strategic direction 1 – Information for focused action: developing a strong strategic information system to understand viral hepatitis epidemics and focus the response; Strategic direction 2 – Interventions for impact: defining essential, high-impact interventions on the continuum of hepatitis services that should be included in health benefit packages; Strategic direction 3 – Delivering for equity: strengthening health and community systems to deliver high-quality services to achieve equitable coverage and maximum impact; Strategic direction 4 – Financing for sustainability: proposing strategies to reduce costs, improve efficiencies and minimize the risk of financial hardship for those requiring the services; Strategic direction 5 – Innovation for acceleration: promoting and embracing innovation to drive rapid progress.
48.	World Health Organization, Regional Office for Africa, 2017 [28]	WHO Africa region	Organization publication	Prevention, Care and Treatment of Viral Hepatitis in the African Region: Framework for Action, 2016–2020.	Framework impact targets: 30% reduction of new cases of chronic viral hepatitis B and C infections, 10% reduction of viral hepatitis B and C related deaths. Framework service coverage targets, include but not limited to: all 47 countries have developed national action plans for the prevention, care and treatment of viral hepatitis, HBV vaccine coverage among infants at 90% region-wide, HBV vaccine coverage among health workers at 90% region-wide, at least 25 countries have introduced a birth dose of hepatitis B vaccine, 20% of people with chronic hepatitis infections diagnosed.

49.	World Health Organization, 2019 [24]	Global	Organization report	WHO position paper, July 2017 — Recommendations.	This position paper replaces the May 2009 WHO position paper on hepatitis B vaccines (Hepatitis B vaccines, 2009). The recommendations emphasize the importance of vaccination of all infants at birth as the most effective intervention for the prevention of hepatitis B virus-associated disease worldwide.
50.	World Health Organization, 2022. [27]	Global	Webpage	Immunization coverage	Hepatitis B vaccine for infants had been introduced nationwide in 190 Member States by the end of 2022. Global coverage with 3 doses of hepatitis B vaccine is estimated at 84%. In addition, 113 Member States introduced nationwide 1 dose of hepatitis B vaccine to newborns within the first 24 hours of life. Global coverage is 45% and is as high as 80% in the WHO Western Pacific Region, while it is only estimated to be at 18% in the WHO African Region.
51.	World Health Organization, 2021 [16]	Global	Organization publication (report)	Global progress report on HIV, viral hepatitis and sexually transmitted infections, 2021. Accountability for the global health sector strategies 2016-2021: actions for impact.	Service coverage targets and progress thus far.<br>Global comparisons of progress in regions.<br>Identification of key populations requiring attention in order to result in improved long-term change in the region (particularly in Africa).
52.	United Nations International Children's Emergency Fund, 2022 [21]	Global: particularly Africa	Webpage	Immunization coverage estimates data visualization 2022	Up to date vaccination coverage data including hepatitis B infant and birth-dose vaccinations.
53.	World Health Organization, 2020 [55]	Global	Organization publication	Prevention of mother-to-child transmission of hepatitis B virus: guidelines on antiviral prophylaxis in pregnancy.	WHO recommends that pregnant women testing positive for HBV infection (HBsAg positive) with an HBV DNA ≥ 5.3 log10 IU/mL (≥ 200,000 IU/mL)1 receive tenofovir prophylaxis from the 28th week of pregnancy until at least birth, to prevent mother-to-child transmission of HBV. This is in addition to three-dose hepatitis B vaccination in all infants, including timely birth dose.
54.	World Health Organization, 2022 [62]	Global / Africa	Webpage	Hepatitis B vaccination coverage	Updated vaccination coverage according to official country estimates and those reported to WHO through the Joint Reporting Form
55.	Njuguna., 2022 [30]	Africa	Conference proceedings<br><br>(Published by Coalition for Global Hepatitis Elimination)	HepB-BD and infant HepB3 coverage status in Africa. Hepatitis B Birth Dose in the African region: Bridging science and advocacy to eliminate mother-to-	Only 15 countries are practicing hepatitis B birth-dose vaccination as part of their national schedule for immunizations. Mauritius uses a selective approach, the rest a universal vaccination approach. The COVID-19 pandemic has impacted the infant vaccinations in the region.

				child transmission of HBV, August 22 2022.	
56.	Kabore, 2021 [29]	Africa	Conference proceedings<br><br>(Published by Coalition for Global Hepatitis Elimination)	Hepatitis B Birth Dose (HepBBD) Vaccination in the WHO African Region. Building community to support the introduction of hepatitis B birth-dose (March 17-18 2021)	Regional targets not met by 2020: only 14 countries adopted universal Hep BBD vaccination, coverage HepB3 73% and only 6% for Hep BBD, prevalence among those under 5 years of age is 2.7 times the global average. Persistent challenges to birth-dose vaccination include the slow progress to uptake by countries and missed opportunities for timely vaccination. There is a need for ongoing generation of evidence and commitments from stakeholders.
57.	Ott et al., 2012 [50]	Global	Systematic review and Modelling study	Global epidemiology of hepatitis B virus infection: New estimates of age-specific HBsAg seroprevalence and endemicity	HBV prevalence was most common in sub-Saharan (SSA) regions of Africa. Western sub-Saharan African countries had some of the highest age-specific HBsAg prevalence in the world reaching up to 12% among children and adolescents in the age-groups up to 19 years in 1990. Although there was a decrease in 2005, the region continued to have high HBV endemicity, which is more pronounced among males. An increase in chronic HBV infection among younger age-groups (0–14 years) occurred in Southern SSA in 2005 compared to 1990 that resulted in age-specific prevalence of 8–9% among young females. Eastern SSA countries faced an increase in the youngest ages and almost no change in other age-groups. In 2005, prevalence peaked at approximately 7% in 0–4 years aged boys and girls and declined with age in this region. A decrease in prevalence was evident in Central SSA which transitioned from high endemicity among younger individuals (age-groups up to 34 years) in 1990 into intermediate endemicity across all ages in 2005.
58.	Keane et al., 2016 [45]	sub-Saharan Africa	Systematic review	Systematic review with meta-analysis: the risk of mother-to-child transmission of hepatitis B virus infection in sub-Saharan Africa.	Compared to Asia, the risk of mother-to-child transmission is low in sub-Saharan Africa. However, the annual number of infants perinatally infected with HBV is twice the number of incident paediatric HIV infections in sub-Saharan Africa (n=190 000). This highlights the importance of preventing mother-to-child transmission of HBV in sub-Saharan Africa, which has been long neglected.
59.	de Villiers MJ et al., 2021 [26]	Global	Modelling study	The impact of the timely birth dose vaccine on the global elimination of hepatitis B	Scaling up timely hepatitis B birth dose vaccination to 90% of new-borns in 110 low- and middle-income countries by 2030 could prevent 710,000 (580,000 to 890,000) deaths in the 2020 to 2030 birth cohorts compared to status quo, with the greatest benefits in Africa. Maintaining this could lead to elimination by 2030 in the Americas, but not before 2059 in Africa.
60.	Boisson et al., 2022 [78]	sub-Saharan Africa	Scoping review	Implementation Approaches for Introducing and Overcoming	Overall conclusions suggested that evidence at the community level was both lacking and pertinent to improved implementation. More specifically recommendations highlight key role players, policy makers and researchers,

				Barriers to Hepatitis B Birth-Dose Vaccine in sub-Saharan Africa	should pay attention to the mother's and the community's role in vaccine uptake.
61.	Bassoum et al., 2020 [61]	sub-Saharan Africa	Systematic review and meta-analysis	Coverage and timeliness of birth dose vaccination in sub-Saharan Africa: A systematic review and meta-analysis.	Birth-dose vaccines achieve acceptable coverage within the first month of life but perform poorly in terms of timely vaccination. Hepatitis B birth-dose vaccine compared to OPV and BCG performs the poorest in term of timely uptake in the region.
62.	Terrault et al., 2016 [56]	Global / America	Organization publication	AASLD guidelines for treatment of chronic hepatitis B.	American Association for the Study of Liver Diseases recommend immunoglobulin and birth-dose vaccination be given preferably within 12 hours to reduce the chance of transmission from >90% to <10%.
63.	Lampertico et al., 2017 [57]	Global / Europe	Organization publication	EASL 2017 Clinical Practice Guidelines on the management of hepatitis B virus infection	European Association for the Study of Liver Diseases states prevention of HBV perinatal transmission, occurring mainly at delivery, is based on the combination of HBIG and vaccination given within 12 h of birth. This prophylaxis reduces the rate of perinatal transmission from >90% to <10%.
64.	Miyahara et al., 2016 [77]	The Gambia	Prospective cohort study	Barriers to timely administration of birth dose vaccines in The Gambia, West Africa	Birth-dose of HBV vaccine coverage at birth at different time points are as follows: 1.1% (117/10851) on day 0–1, 5.4% (586/10851) on day 7, 58.4% (6340/10851) on day28, and 93.1% (10,100/10,851) and 93.6% (10,162/10,851) at 6 months and 12 months respectively. Factors found to be associated with delayed vaccination: living in urban and peri-urban settings, long distance from vaccination delivery points, Fula ethnicity, and low maternal education.
65.	Schweitzer et al., 2017 [82]	Global	Cross-sectional study	Hepatitis B vaccination timing: results from demographic health surveys in 47 countries.	The substantial inequities in the implementation and adherence to national immunization schedules for hepatitis B vaccine underscore the continued need for strengthening immunization systems. Timing is suggested as being incorporated as a performance indicator to complement coverage assessments.
66.	World Health Organization, 2007 [88]	n/a<br>Global	Organization Publication	Everybody's business: Strengthening health systems to improve health outcomes: WHO's framework for action	According to the WHO, a health system encompasses "all organizations, people and actions whose primary intent it is to promote, restore and maintain health". The WHO provides a health systems framework consisting of six dimensions (service delivery; health workforce; information; medical products, vaccines, and technologies; financing; leadership and governance) commonly referred to as building blocks and their overall goals or outcomes
67.	Mounier-Jack et al., 2014 [31]	n/a<br>Reference to low- and middle-income countries	Debate	Measuring the health systems impact of disease control programmes: A critical reflection on the WHO	Reflecting on the strengths and weaknesses of the WHO health systems framework. The framework provides a good "language" to discuss health systems issues across a broad scope of role-players, however, it has the effect of reducing the health system to a complicated and not a complex system with dynamic inter-linked system components.

				building blocks framework	
68.	Travis et al., 2004 [32]	Low- and middle-income countries	Review	Overcoming health-systems constraints to achieve the Millenium Development Goals	Health systems strengthening is a vital part of improvement in population health and health outcomes. Examples presented are from those used to address the MDGs. What is particularly highlighted is that fragmented or weak health systems are nearly incapable of implementing certain interventions effectively. Research into health systems strengthening will have far reaching effects.
69.	Amponsah-Dacosta et al., 2020 [93]	sub-Saharan Africa	Systematic review	Health systems constraints and facilitators of human papillomavirus immunization programmes in sub-Saharan Africa: A systematic review.	Development of a logic model used in health systems approach to immunization programs. The logic model combines the 8 components of routine immunization with the 6 WHO building blocks resulting in 6 cross cutting themes, namely: (i) the governance and policy landscape; (ii) the capacity of the health workforce; (iii) the availability of potent vaccines, cold chain and logistics systems; (iv) the quality of health service delivery; (v) the state of health information and community partnerships; and (vi) the availability of equitable and sustainable health financing
70.	Rohwer et al., 2017 [92]	Low- and middle-income countries<br><br>sub-Saharan	Methods paper	Series: Clinical Epidemiology in South Africa. Paper 3: Logic models help make sense of complexity in systematic reviews and health technology assessments	Two distinct templates are presented: the system-based logic model, describing the system in which the interaction between participants, intervention, and context takes place; and the process-orientated logic model, which displays the processes and causal pathways that lead from the intervention to multiple outcomes. In particular the system-based logic model helps to identify sources of heterogeneity and complexity.
71.	Petticrew, 2011 [36]	n/a	Editorial	When are complex interventions 'complex'? When are simple interventions 'simple'	Expanding on the definition of is what is considered a simple or complex intervention. Adopting a simple or complex perspective to the research question would provide a simple or complex explanation to the health problem. This can help focus the intention of the research and advise the methods.
72.	Petticrew et al., 2013 [38]	n/a	Methods paper	Complex interventions and their implications for systematic reviews: A pragmatic approach	Many sources of complexity exist, broadly they can be found as a characteristic of the intervention or the intervention's causal pathway. Challenges in demonstrating a complex perspective exist, this paper provides an approach to thinking about sources of complexity in interventions, and how this can be mapped onto specific types of study. Noteworthy is the fact that even simple interventions can have complex causal effects when contextual factors are considered.
73.	Shiell et al., 2008 [33]	n/a	Analysis report	Complex interventions or complex systems? Implications for health economic evaluation	Evaluation of complexity as a property of an intervention and that of the system and its effect on economic evaluation. We need to recognise whether we have a complex intervention or an intervention in a complex system, and whether the dynamic characteristics of the system matter enough for us to

					change our evaluation approach. Developing a means of "diagnosing" complexity is particularly important.
74.	Murray et al., 2000 [90]	n/a	Theme paper	A framework for assessing the performance of health systems	Framework for assessing health systems developed for the World Health Organization in the attempts to improve health systems performance.
75.	Shen et al., 2014 [94]	Low resource settings	Commentary	The future of routine immunization in the developing world: Challenges and opportunities	Established a framework that describes the critical elements of routine immunization programs. Eight core components are identified, these include: (i) policy, standards and guidelines; (ii) strong governance, management and organization; (iii) human resources for health; (iv) vaccine cold-chain and logistics; (v) quality and reliable service delivery; (vi) communication and community partnerships; (vii) generation and use of quality immunization data; (viii) sustainable vaccine and immunization financing
76.	World Health Organization, 2017 [63]	Global	Organization publication	Global Hepatitis Report 2017	Expands on the burden of disease and area's needing critical attention, the strategies in place and the strategies yet to be implemented in certain areas towards elimination of hepatitis.
77.	Langlois, 2018 [35] (World Health Organization)	n/a	Organization publication (Methods paper)	Evidence synthesis for health policy and system: a method guide	Performing reviews of complex health policy and systems interventions: method guide on how to address complexity in HPSR and in particular in review research
78.	Hawe et al., 2009 [39]	n/a	Literature review	Theorising interventions as events in systems	Shifting the focus to the interaction between intervention and context could provide an explanation for outcomes, owing to the complexity created by this interaction. Drawing on social network analysis and complex systems theory, the impact of interventions on evolving networks like relationships or displacing existing activities and redistributing or transforming resources might be a useful tool in designing more effective interventions.
79.	Breakwell et al., 2022 [67]	Sierra Leone	Cross-sectional study	Assessing the Impact of the Routine Childhood Hepatitis B Immunization and the Need for Hepatitis B Vaccine Birth Dose in Sierra Leone, 2018	Despite coverage of 77%-85% for infant hepatitis vaccinations (Hep B3) persistence of HBsAg among children was detected. Those born to HBsAg positive mothers had a HBsAg prevalence of 5.9% compared to HBsAg negative mothers (0.7%). The need for HepB-BD for further protection is needed together with improved commitment to infant hepatitis B vaccinations.
80.	Kruk et al., 2008 [86]	Developing countries	Literature review	Assessing health system performance in developing countries: A review of the literature	Most commentators agree that a well-performing health system is effective, equitable, and efficient. The literature presented suggests the breadth of indicators available for measuring performance of component. These components included equity, effectiveness, health status, patient satisfaction, access, efficiency.

81.	de Savigny et al., 2009 [34]	n/a<br>useful for LMIC	Organization publication	Systems thinking for health systems strengthening	Defining health systems, systems thinking and health systems strengthening interventions.
82.	Van Olmen et al., 2012 [91]	n/a<br>useful for LMIC	Literature review<br>Historical analysis for framework development	The health system dynamics framework: the introduction of an analytical model for health system analysis and its application to two case-studies	The development of a health systems dynamic framework encompassing the context and population as part of the health system, interacting with the resources, leadership and governance impacting service delivery and eventual outcomes and goals.
83.	Arah et al., 2003 [89]	High income countries<br>Organizations: WHO and OECD	Review of cases (countries framework performance)	Conceptual frameworks for health systems performance: A quest for effectiveness, quality, and improvement	Comparing national and organization frameworks. These performance measurement and management frameworks still need to address some conceptual and operational issues concerning effectiveness and quality. Effectiveness and quality need to better translate quality and effectiveness at health service level and health systems level. Indicators of effectiveness should be clearly linked to realistic, pre-defined, system-wide targets or outcomes.
84.	World Health Organization, 2021 [17]	Global and regional	Organization publication (Report)	Web Annex 1. Key data at a glance. In: Global progress report on HIV, viral hepatitis and sexually transmitted infections, 2021. Accountability for the global health sector strategies 2016-2021: actions for impact	Up to date global and regional figures on hepatitis (and HIV and STIs) prevalence. Alerting the African region to the high ≥5 years of age prevalence of chronic hepatitis B infections despite ongoing strategies to curb the epidemic. There are 296 million [228–423 million] people living with chronic hepatitis B virus infection, 6 million [4–11 million] children younger than five living with hepatitis B virus infection. 7.5% prevalence of HBV in the general population, 2.5% children younger than 5years of age equating to 4 300 000 children. Only the Western Pacific region has a higher amount of chronic HBV sufferers than the African region.
85.	Craig et al., 2008 [37]	n/a	Research methods and report	Developing and evaluating complex interventions: the new Medical Research Council guidance	Guidance on Medical Research Council framework for complex interventions. Many issues surrounding evaluation of complex interventions are still debated, that methods will continue to develop, and that practical applications will be found for some of the newer theories.
86.	Samb., et al [87]	Global	Review and report	An assessment of interactions between global health initiatives and country health systems	Several findings useful: encouragement of health systems strengthening at the speed and motivation that existed when starting up global health initiatives (GHI), agree on indicators for health system strengthening, improve the alignment of planning processes and resource allocation among GHI and between GHIs and countries, generate reliable data for the costs and benefits of strengthening, ensure a rise in national and global health funding ensuring more predictable financing to ensure sustainable and equitable growth of health systems.

87.	Maponga et al., 2017 [20]	Low- and middle-income countries	Debate (pre-conference meeting)	Highlights from the 3rd International HIV/Viral Hepatitis Co-Infection Meeting - HIV/Viral Hepatitis: Improving Diagnosis, Antiviral Therapy and Access	Discussions concluded the increased efforts towards universal implementation of birth dose vaccination for hepatitis B without further delay; sustainable access to antiviral therapy for HBV mono-infected individuals; increased diagnosis and treatment of viral hepatitis, particularly HCV among people using injectables drugs; end of stigmatization of people living with HIV and/or viral hepatitis.
88.	Gueye, 2016 [54]	Senegal	Cross-sectional	HBV carriage in children born from HIV-seropositive mothers in Senegal: The need of birth-dose HBV vaccination	Study showed a high rate of HBsAg prevalence in children less than 24 months old in Senegal. HBsAg prevalence was 3.6 times lower among children born from mother under 3TC/TDF-based prophylaxis, showing the protective effect of antiretrovirals on MTCT.
